# Spatiotemporal dynamics of forest ecosystem carbon budget in Guizhou: customisation and application of the CBM-CFS3 model for China

**DOI:** 10.1186/s13021-022-00210-0

**Published:** 2022-07-02

**Authors:** Yuzhi Tang, Quanqin Shao, Tiezhu Shi, Zhensheng Lu, Guofeng Wu

**Affiliations:** 1 MNR Key Laboratory for Geo-Environmental Monitoring of Great Bay Area & Guangdong Key Laboratory of Urban Informatics & Shenzhen Key Laboratory of Spatial Smart Sensing and Services, Shenzhen University, Shenzhen, 518060 China; 2 Key Laboratory of Land Surface Pattern and Simulation, Institute of Geographic Sciences and Natural Resources Research, Chinese Academy of Sciences, Beijing, 100101 China; 3 School of Architecture and Urban Planning, Shenzhen University, Shenzhen, 518060 China; 4 Beijing Xiaomi Technology Co., Ltd, Beijing, 100085 China

**Keywords:** Forest carbon dynamics, Disturbances, Customisation, CBM-CFS3, China

## Abstract

**Background:**

Countries seeking to mitigate climate change through forests require suitable modelling approaches to predict carbon (C) budget dynamics in forests and their responses to disturbance and management. The Carbon Budget Model of the Canadian Forest Sector (CBM-CFS3) is a feasible and comprehensive tool for simulating forest C stock dynamics across broad levels, but discrepancies remain to be addressed in China. Taking Guizhou as the case study, we customised the CBM-CFS3 model according to China’s context, including the modification of aboveground biomass C stock algorithm, addition of C budget accounting for bamboo forests, economic forests, and shrub forests, improvement of non-forest land belowground slow dead organic matter (DOM) pool initialisation, and other model settings.

**Results:**

The adequate linear relationship between the estimated and measured C densities (*R*^2^ = 0.967, *P* < 0.0001, *slope* = 0.904) in the model validation demonstrated the high accuracy and reliability of our customised model. We further simulated the spatiotemporal dynamics of forest C stocks and disturbance impacts in Guizhou for the period 1990–2016 using our customised model. Results shows that the total ecosystem C stock and C density, and C stocks in biomass, litter, dead wood, and soil in Guizhou increased continuously and significantly, while the soil C density decreased over the whole period, which could be attributed to deforestation history and climate change. The total ecosystem C stock increased from 1220 Tg C in 1990 to 1684 Tg C in 2016 at a rate of 18 Tg C yr^−1^, with significant enhancement in most areas, especially in the south and northwest. The total decrease in ecosystem C stock and C expenditure caused by disturbances reached 97.6 Tg C and 120.9 Tg C, respectively, but both represented significant decreasing trends owing to the decline of disturbed forest area during 1990–2016. Regeneration logging, deforestation for agriculture, and harvest logging caused the largest C stock decrease and C expenditure, while afforestation and natural expansion of forest contributed the largest increases in C stock.

**Conclusions:**

The forests in Guizhou were a net carbon sink under large-scale afforestation throughout the study period; Our customised CBM-CFS3 model can serve as a more effective and accurate method for estimating forest C stock and disturbance impacts in China and further enlightens model customisation to other areas.

**Supplementary Information:**

The online version contains supplementary material available at 10.1186/s13021-022-00210-0.

## Background

Global climate emergencies are generally recognised as climate change intensifies and becomes widespread and rapid [1–3]. China, one of the largest carbon (C) emitters of the world, has promised to peak carbon dioxide (CO_2_) emissions by 2030 and achieve carbon neutrality by 2060 [4, 5]. One of the essential methods to fulfil this commitment is to grow and maintain forests [6, 7]. Serving as an important “natural solution” to climate change, forest ecosystems play a key role in C sequestration in the terrestrial biosphere, while the latter provides a net sink for ∼20% of anthropogenic greenhouse gas (GHG) emissions [8, 9]. As countries such as China seek to understand and influence the trajectory of global change, they require suitable monitoring and modelling approaches to predict current and future C stock dynamics in forests and their response to disturbance and management [10, 11].

The substantial models used to quantify forest C stocks can be classified into two groups: (i) process-based models driven by photosynthesis simulations and other ecological processes, and (ii) empirical models driven by forest inventories and empirical growth data [12, 13]. Typically, process-based models focus more on the simulation of vegetation photosynthesis and productivity [14–16], and the potential effects of climate change [17–19], with less concern about the C stock changes in dead organic matter (DOM) and those caused by natural and anthropogenic disturbances. In addition, process-based models are usually complex and require detailed measurements of leaf area index, climate variables, and soil variables [10], which are unavailable for many areas [12, 20]. On the other hand, empirical models, such as EFISCEN [21], CO2FIX [22], FORMICA [23], and CBM-CFS3 [23], consider C dynamics in various forest C pools while incorporating the impacts of disturbances and forest management into forest C simulations, thus providing detailed C budget information and decision support for the scientific management of forest ecosystems. Moreover, empirical models are better suited than process-based models for using data collected from small-scale investigations in plots in the field or from large-scale surveys at the regional and national levels [20].

Among the empirical models, the Carbon Budget Model of the Canadian Forest Sector (CBM-CFS3) is considered the most comprehensive model. It can be applied at the stand, landscape, and national levels, while CO2FIX can only treat stand-level areas, and EFISCEN is suitable for landscape-level areas [12]. CBM-CFS3 focuses on C dynamics with diverse disturbances and land use and land cover (LULC) changes, while EFISCEN only includes climate change, clear-cut logging, and thinning, and FORMICA only considers clear-cut logging and thinning [23]. In addition, CBM-CFS3 yields non-equilibrium soil conditions that reflect changes in disturbance regime, management, or species relative to historical conditions to initialise soil pools, while the other models use observed C stocks to initialise in the absence of disturbance [10]. Moreover, only CBM-CFS3 accounts for merchantability and more specific C pools and provides abundant default parameters related to tree growth, biomass, and DOM C estimation, and hundreds of default disturbance matrices for quantifying the impacts of various disturbances, which is not the case in other models. Therefore, CBM-CFS3 represents efficiency, reliability, and convenience in simulating forest C dynamics, which contribute to its broad application. Hitherto, CBM-CFS3 has been applied in many countries in the Northern Hemisphere, including Canada [11, 24, 25], Italy [13], South Korea [20], Mexico [26], Russia [27], and Slovenia [28]. Additionally, CBM-CFS3 has also been parameterised and employed in forest C dynamics simulations for most European Union countries [29, 30].

The CBM-CFS3 has been applied in China, mostly regionally [31–35]. However, despite the specific parameterisation to the local context, these studies failed to consider the inconformity between the model applicable condition and the actual situation in China, which cause misestimation to some extent. Located in the East Asian monsoon climate zone, the climate, ecological environment, and vegetation in China differ significantly from those in North America and Europe. More importantly, there is a discrepancy between the forest inventory data in China and Canada, which requires modification of biomass C algorithms to adapt to the Chinese forest inventory system. Furthermore, the current CBM-CFS3 only calculates the C stock in arbour forests; while, in China, there are vast bamboo, economic, and shrub forests, therefore the C stocks in these forests should not be neglected. Additionally, some oversimplified designs, for example, setting the C content of all tree species as 0.5 g C/g dry matter, of the model are too rough to be applied in regional-scale estimation.

The first objective of this study was to customise the CBM-CFS3 model according to China’s context, thus enhancing its adaptability and accuracy in forest C budget estimation in China. To achieve this goal, we employed the Guizhou Province, which is of great importance to the national forest carbon sink function, as our study area to implement the customisation, parameterisation, and application of the CBM-CFS3 model. Our second objective was to analyse the spatiotemporal dynamics of the forest ecosystem C budget in Guizhou, as well as the disturbance impacts for the period 1990–2016, therefore sharing lessons learned from the Guizhou forestry history for forest management, and further examine the applicability and reliability of our customised model. This study is expected to serve as an important reference for studies on forest ecosystem C budget simulations in China and further enlighten model customisation in other areas.

## Materials and methods

### The Carbon Budget Model (CBM-CFS3)

The CBM-CFS3 model is an inventory-based, yield-data-driven model that simulates the dynamics of forest C stocks at the stand, landscape, and national levels [10, 13]. Implementing the Tier 3 approach of the Intergovernmental Panel on Climate Change (IPCC) Good Practice Guidance (GPG) reporting standards [36], the model uses the “one inventory plus change” method, which requires detailed data such as forest inventory, natural disturbance events, forest management activities, LULC changes (LUCC), and ecological parameters associated with forest growth, biomass turnover, litterfall, transfer, and decomposition [10]. The CBM-CFS3 can quantify past annual forest C stocks and stock changes caused by ecological processes and disturbances and simulate future forest C dynamics under different scenarios to assess policy and management alternatives. The model is not spatially explicit, but with the use of spatially referenced IDs of forest stands and GIS tools, the spatiotemporal dynamics of forest C stocks and stock changes can be simulated conveniently. All five GPG forest ecosystem C pools [36], that is, aboveground (AG) and belowground (BG) biomass, and DOM, including litter, dead wood, and soil organic matter (SOM), which were further divided into 10 biomass and 11 DOM C pools in the CBM-CFS3[10], were used for estimation.

When the model starts running, a simulation initialisation boots to populate the biomass and DOM C stocks for each stand. Non-equilibrium soil conditions are yielded to reflect the changes in the disturbance regime, management, or species at the start of the simulation, relative to historical conditions [10]. In each annual time step, the CBM-CFS3 first utilises a library of yield tables to estimate the gross merchantable volume in the absence of natural disturbances and management practices, and then applies volume-to-biomass equations and multinomial logit models [37] to transform the merchantable volume into AG biomass (AGB) in all components of a stand. Empirical equations developed by Li et al. [38] are used to derive BG biomass (BGB) from AGB. Different portions of a stand are split and assigned to corresponding biomass pool, i.e. merchantable + bark, other wood + bark, foliage, coarse roots, and fine roots [10]; a fixed C content of 0.5 g C/g dry matter is used to convert units of dry matter to mass of C. After growth simulation, the CBM-CFS3 uses annual biomass turnover rates and litterfall rates to estimate the biomass turnover and the transfer of dead biomass to one or more DOM pools, respectively. Each biomass pool is assigned a specific turnover rate, and the organic matter therein only transfer to related DOM pools in relatively stable proportions [10, 39]. Finally, a temperature-dependent decay rate is applied to model the decomposition of every DOM pool, and empirical proportions are used to determine the decomposed C stock released to the atmosphere or transferred to a more stable slow DOM pool. If natural or anthropogenic disturbances occur, the CBM-CFS3 exploits a suite of disturbance matrices to simulate the impact on forest C stocks, including the proportion of C transferred between pools, as fluxes to the atmosphere, and as transfers to the forest products sector, which vary significantly depending on disturbance type. Post-disturbance C dynamics and LUCC impact are also carefully considered in the year following the disturbance event. For detailed model descriptions, algorithms, and parameter settings, see Ref. [10] and [40].

### The Forest Resource Planning and Design Survey (FRPDS) data

The FRPDS is a thorough regional forest resource inventory based on county administrative areas or forest management units (such as state-owned forest farms, nature reserves, and forest parks), initiated by the Chinese provincial forestry bureaus for forest management needs. The FRPDS divides regional forests into a mass of stands according to similar dominant tree species (group), age group, canopy density, site conditions, forest origin, forest land type, and wood ownership, thus presenting fundamentally the same internal characteristics within each stand.

During 2015–2016, Guizhou Province conducted the fourth FRPDS (FRPDSGP). More than a hundred stand attributes and site conditions over three million stands were recorded, including land-use type, forest land type, plant type, dominant tree species, average age, age class/group, stand volume per hectare, stand area, origin, soil type, community structure, disaster class, health class, and so on. The classification of some survey items is presented in Table 1. The FRPDSGP was archived in the ArcGIS Geodatabase.gdb format; each stand was documented as a record (i.e. a polygon), and every stand attribute or site condition was set as a field. Details of the investigation can be found in Tang et al. [41].

**Table 1:** Classification of some survey items in the Forest Resource Planning and Design Survey (FRPDS)

Survey items	Classification	Description
Land-use type	Forest land	Areas for forestry ecological construction, production and management, with a minimum area of 667 m^2^
	Cropland	Cultivated land, farmland, includes two sub-classes: paddy and dryland
	Grassland	Pasture, rangeland, grassland
	Inland water and wetland	Lakes, rivers, reservoirs, wetland, and other water bodies
	Built-up land	Areas for residential, industrial, commercial, mining, traffic and transport, tourist facilities, parking sites, gardens and parks
	Bare land	Unused and unproductive land surface with vegetation coverage ≤ 5%
Forest land type	Arbour forest land	Forest land composed of arbour species, with a crown density ≥ 20%; or a crown density < 20% but retention rate ≥ 80% with steadily growing young trees in planted stands
	Sparse forest land	Forest land composed of arbour species, with a crown density of 10–19%
	Bamboo forest land	Forest land composed of bamboos with a minimum diameter at breast height (DBH) of 2 cm
	Shrub forest land	Forest land with a minimum crown cover of shrub species of 30%; includes two sub-classes: special shrubs and general shrubs
	Other forest land	Unclosed afforestation land, nursery land, clear-cut land, burned forest land, and planned forest land
Plant type	Arbour species	Trees that have a distinct trunk, with tree height > 5 m and DBH > 5 cm at maturity; includes three sub-classes: coniferous species (i.e. softwoods), broad-leaved species (i.e. hardwoods), and mixed tree species
	Bamboo species	A kind of tall tropical plants with hard, hollow stems
	Economic plant species	Arbours and shrubs mainly for the production of fruit, edible oil, drinks, flavourings, industrial raw materials, and medicinal materials
	Shrub species	Large plants that have several woody stems coming from the ground, in addition to the economic shrub species
Age class	–	Classification for tree or stand age by a certain number of years according to the forest management requirements and biological characteristics of tree species, denoted by roman numerals I, II, III, IV, V, etc., from young to old; the number of years included in each age class is called the age class period
Age group	Young	Stand ages that at the youngest stage in tree growth, with stand age in age class I or I–II; the growth of trees is slow in their infancy and increases rapidly after canopy closure
	Middle-aged	Stand ages that have reached the age class lower one or two age class periods than the near-mature age group; trees grow vigorously in diameter, with flowering and fruiting
	Near-mature	Stand ages that have reached the age class lower one age class period than the mature age group; trees slowdown in growth and are close to be maturely utilised
	Mature	Stand ages that have reached the age class or one age class period older for harvest; trees are in full maturity and are able to be harvested
	Post-mature	Stand ages that have reached the age class older than the mature age group; trees are being senescent

### Case study area: Guizhou, China

**Fig. 1:**
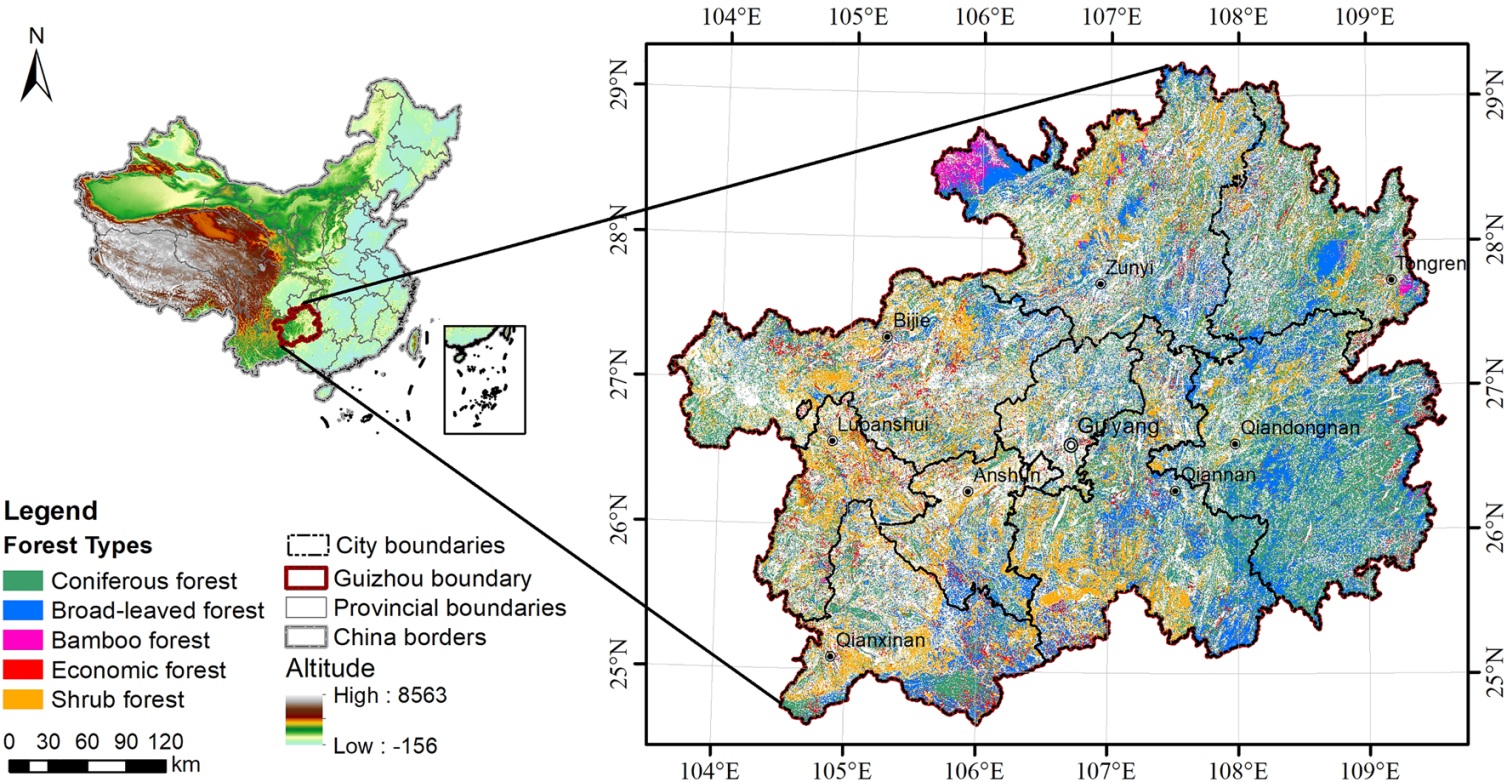
Location and forest types spatial distribution of Guizhou

Guizhou (24°37–29°13 N, 103°36–109°35 E) is a province located in southwest China and the eastern slope zone of the Yunnan-Guizhou Plateau (Fig. 1). It is composed of ten cities: Guiyang (the capital city), Liupanshui, Zunyi, Anshun, Bijie, Tongren, Qiandongnan, Qianxinan, Qiannan, and Guian, covering a total area of 56,000 km^2^. The terrain consists mainly of mountains and hills, with a mean altitude of approximately 1100 m. Karstification is highly developed in this area, and karst types are the most diverse of any karst area in the world [42]. Dominated by a typical subtropical humid monsoon climate, this area has an average annual temperature of 15 °C and average annual rainfall of 1177 mm. Superior geographic and climatic conditions contribute to abundant forest resources in Guizhou, including coniferous, broad-leaved, and bamboo forests [43, 44]. Unfortunately, this fragile karst region has suffered severe rocky desertification owing to long-term and large-scale deforestation and reclamation in the last century, leading to extensive degradation of forests to shrubs [41]. It was not until 2000 a series of ecological restoration programs, such as the Grain for Green Program, were implemented in the area that the situation was ameliorated [45, 46]. To restore vegetation and develop the economy simultaneously, economic forests for producing fruit, medicine, edible, and industrial raw materials were largely grown in the area. The fourth FRPDSGP in 2016 demonstrated that the forest area (10,761,950 ha) covered 61.09% of Guizhou, of which 39.7% was coniferous forests, 27.6% was broad-leaved forests, 25.2% was shrub forests, 6.0% was economic forests, and 1.5% was bamboo forests (Fig. 1). The Chinese fir (*Cunninghamia lanceolata* [Lamb.] Hook.) and Masson pine (*Pinus massoniana* Lamb.) are the major tree species in Guizhou, occupying 15.75% and 15.65% of the total forest area, respectively; notwithstanding, other tree species, such as cypress (*Cupressus funebris* Endl.), oak (*Quercus* spp.), cyclobalanopsis (*Cyclobalanopsis* spp.), and birch (*Betula* spp.) also cover large areas of the study area. Detailed information about the main tree species is provided in the Additional file 1: Part I.

### Customisation of the CBM-CFS3 for Guizhou, China

#### Customisation of the aboveground biomass C stock algorithm

**Fig. 2:**
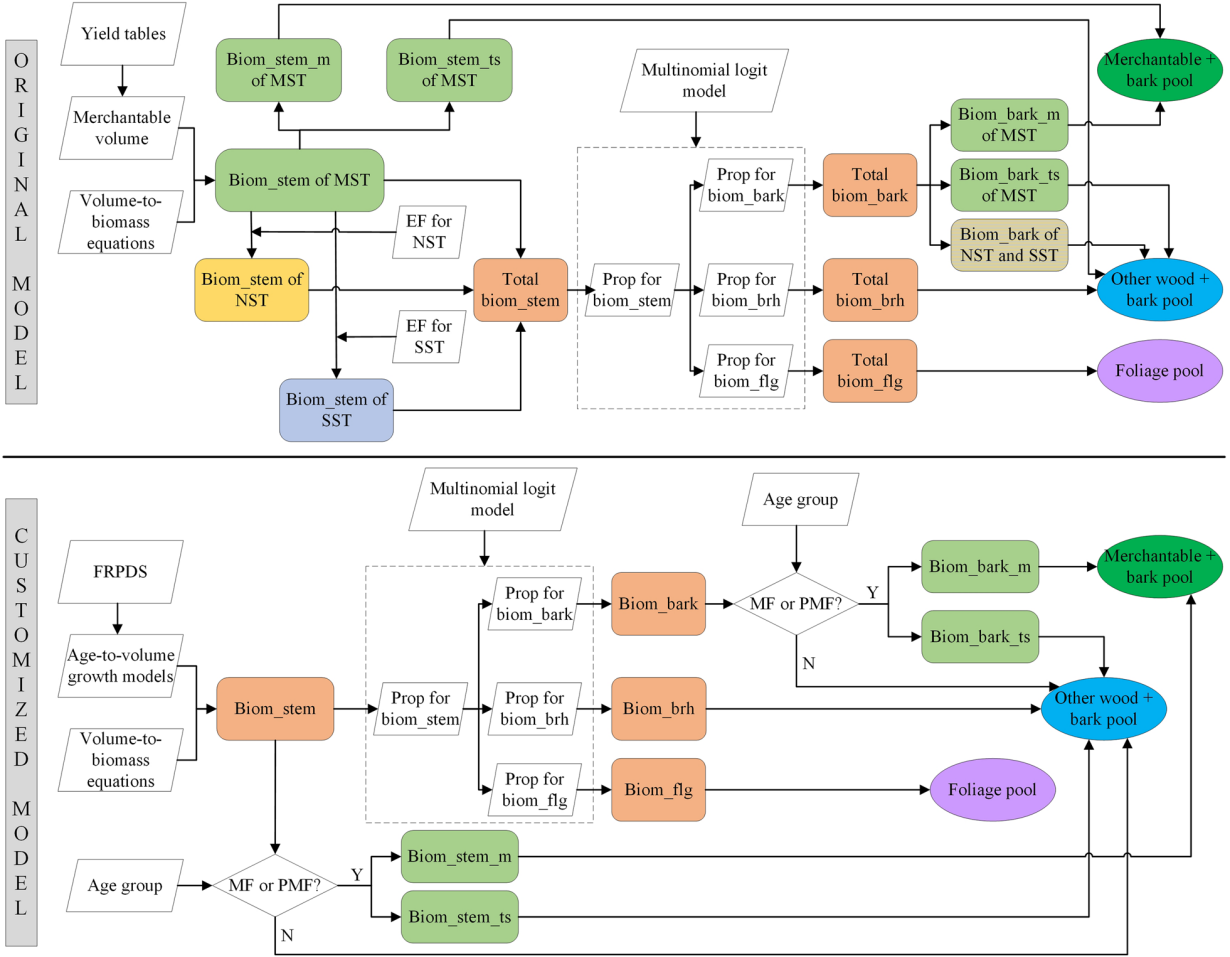
Comparison between the aboveground (AG) biomass carbon (C) stock algorithm workflows of original model and customised model. In the figure, MST = merchantable-sized trees, NST = nonmerchantable-sized trees, SST = sapling-sized trees, biom_stem = biomass in stemwood, biom_bark = biomass in stem bark, biom_brh = biomass in branches, biom_flg = biomass in foliage, biom_stem_m = biomass in merchantable portion of stemwood, biom_stem_ts = biomass in tops and stumps portion of stemwood, biom_bark_m = biomass in merchantable portion of bark, biom_bark_ts = biomass in tops and stumps portion of bark, EF = expansion factor, prop = proportion, MF = mature forests, and PMF = post-mature forests

The CBM-CFS3 employs a hierarchical set of equations developed by Boudewyn et al. [37] to estimate AGB C stocks. Boudewyn et al. [37] assumed that the biomass in a particular forest stand could be summarised into three discrete stand components: merchantable-sized trees (MST), non-merchantable-sized trees (NST), and sapling-sized trees (SST). They then estimated the stemwood biomass of MST using yield tables in units of merchantable volume and volume-to-biomass equations, and then calculated the stemwood biomass of NST and SST through the stemwood biomass of MST and their fitted expansion factors. The biomass in stem bark, branches, and foliage for live trees of all sizes was derived from the stemwood biomass estimates, according to their proportions in the total tree which were fitted simultaneously using a multinomial logit model. Subsequently, the biomass in merchantable portions of stemwood and stem bark of MST were assigned to “merchantable + bark” pool, while the biomass in tops and stumps portion of stemwood and stem bark of MST, the biomass in stemwood and bark of NST and SST, and all branches were assigned to the “other wood + bark” pool (Fig. 2).

The CBM-CFS3 divides a forest stand into three discrete stand components for the following reasons: first, it assumes that a sample plot contains trees in different growth stages and different diameter classes; second, it uses yield tables to represent the growth of merchantable wood volume, which can only estimate the stemwood biomass of MST; thus, additional calculations are required to derive the stemwood biomass of NST and SST. However, this was not the case for the FRPDS in China. According to “The Forest Resource Planning and Design Survey (FRPDS) data” section, the spatial units of the FRPDS, that is, stands, have basically the same internal characteristics; thus, it is assumed that there are no significant differences in tree ages and diameter classes within one stand. As the FRPDS records the stand volume per hectare and stand age of all stands that have reached the initial diameter at breast height (DBH) (5 cm), the stand volume of all growth stages can be estimated by building a hierarchical set of age-to-volume growth models (see Ref. [41]). Therefore, the calculations of biomass for live trees of all sizes in a stand are the same by using the same set of age-to-volume growth models and volume-to-biomass equations, and are therefore significantly different from the original model. Additionally, the stand age group can be used to assign the biomass in different stands and tree portions to the corresponding biomass pools. As shown in Table 1, the FRPDS classifies forests into five age groups: young forests (YF), middle-aged forests (MAF), near-mature forests (NMF), mature forests (MF), and post-mature forests (PMF). The stands of YF can be regarded as SST, while those of MAF and NMF can be regarded as NST, and those of MF and PMF can be regarded as MST, in accordance with their definitions. Consequently, after the biomass estimation for each tree proportion, the biomass in merchantable portion of stemwood and stem bark of MF and PMF are assigned to “merchantable + bark” pool; the biomass in tops and stumps portion of stemwood and stem bark of MF and PMF, the biomass in stemwood and bark of NMF, MAF and YF, and all branches are assigned to the “other wood + bark” pool, which are consistent with the original model (Fig. 2).

#### Carbon budget accounting for bamboo forests, economic forests and shrub forests

The CBM-CFS3 is not designed to estimate the C budget in bamboo forests, economic forests, and shrub forests, which are not the main forest types in Canada. A review of studies carried out in China indicates that bamboo is a relatively important carbon store [47–49], and shrub forests and economic forests are widely distributed in China, for example, covering 25% and 6% of Guizhou’s forest area, respectively; the C stocks in these three forest types should not be neglected. However, the FRPDSGP did not record the stand volume of bamboo forests, economic forests, and shrub forests because their plant morphological characteristics are considerably different from those of the arbour species and could not be measured by the usual method based on DBH and trunk [50]. In other words, their C stock cannot be estimated through age-to-volume and volume-to-biomass equations; therefore, a new methodology needs to be established.

**Fig. 3:**
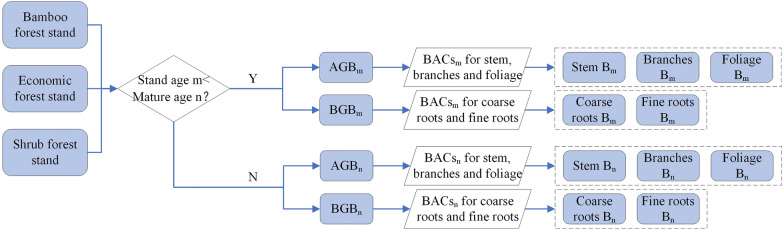
Workflow for estimating AG and belowground (BG) biomass per unit area (biomass_pua_) in bamboo forests, economic forests and shrub forests. In the figure, AGB = AG biomass_pua_, BGB = BG biomass_pua_, BACs = biomass allocation coefficients, B = biomass_pua_, the lower right subscripts at the AGB, BGB, BACs and B represent the stand age

In China, relevant studies have revealed that the biomass in bamboo forests, economic forests, and shrub forests generally remain the same after a short period of rapid early growth, and can thus be estimated by multiplying their forest areas by biomass per unit area (biomass_pua_) [51–53]. For instance, an individual Moso bamboo (*Phyllostachys heterocycla cv. Pubescens*) can complete its early growth within one year and reach maturity within six years, then its biomass_pua_ grows no more afterwards [54]. This method is simple and feasible; however, it overlooks the significant biomass_pua_ increase in early growth, which could cause misestimations. Herein, we propose to estimate the biomass_pua_ before maturity by establishing an age-biomass_pua_ series. The AG and BG biomass_pua_ in different forest types at different ages before maturity were collected from relevant studies and then assigned to stem, branches, foliage, coarse roots, and fine roots according to the collected biomass allocation coefficients for each proportion at associated age. Biomass_pua_ at- and after- maturity remained unchanged (Fig. 3). After the biomass_pua_ estimation, the total biomass C of a stand can be obtained by multiplying the biomass_pua_ by its stand area and the relevant C content. The biomass turnover rates and litterfall rates of bamboo forests, economic forests, and shrub forests are assigned to those of evergreen broad-leaved forests, and the calculation of the DOM C of the three forest types follows the DOM C algorithm as arbour forests.

#### Improvement of non-forest land belowground slow DOM pool initialisation

Plant litter materials provide the primary resources for organic matter formation in soil [55], and human activities related to land use, such as cultivation, influence the organic matter input and the ability to sequester carbon in soil [56, 57]. The CBM-CFS3 sets the default initial values according to soil type for C stocks in the BG slow DOM pool, one of the SOM pools, on non-forest land prior to afforestation [10, 40]. However, soil type failed to reflect vegetation change and human impact on SOM. When LUCC occurs, for example, converting grassland to cropland, the soil type remains the same, but the BG slow C stock will change slowly over time. To address this deficiency, we set the default initial values for BG slow C stocks on non-forest land considering the “vegetation type + LULC” classification, in which the non-forest land is classified into paddy, dryland, grassland, wetland, inland water, built-up land, and bare land.

Accordingly, we modified the BG slow C stock changes estimation as below: During the first 20 years after LUCC occurs, the BG slow C stock of an original non-forest land soil gradually decrease or increase to that of the latter non-forest land soil, and then remain unchanged, which is in accordance with the United Nations Framework Convention on Climate Change (UNFCCC)^1^ [58] and Kurz et al. [10]; if the non-forest stand is afforested during the 20 years, then its BG slow C stock starts to change from that year in line with the forest stand BG slow DOM pool algorithm of CBM-CFS3.

#### Customisation and improvement of other model settings

The CBM-CFS3 classifies land-use according to the UNFCCC land-use category, which documents whether and which type of land-use conversion associated with forest has occurred, to account for the LUCC impact on forest C stocks. The UNFCCC land-use category combines the land-use conversion type and a transition period (the default assumption is 20 years) after conversion to determine the BG slow C stock changes; however, we have considered this in “Improvement of non-forest land belowground slow DOM pool initialisation” section. Therefore, we applied the land-use type classification of FRPDSGP (Table 1) directly instead of the UNFCCC land-use category. By comparing the land-use type before and after a given year in a stand, we can determine whether and which type of LUCC has occurred, and thus account for the forest C stock change of the stand accordingly.

Moreover, we improved the initial value setting of C content and annual biomass turnover rates. The CBM-CFS3 sets the C contents of all tree species as 0.5 g C/g dry matter, and assigns the annual biomass turnover rates by ecological regions, which are too rough to be applied in regional-scale estimation. To improve accuracy, we set the C contents by tree species and the annual biomass turnover rates of different biomass C pools according to vegetation type and tree portions simultaneously. The C content values ranged from 0.439 to 0.598, and the annual biomass turnover rates ranged from 0.017 to 0.952, showing the significant differences in C contents and biomass turnover rates among tree species and tree portions and the necessity to differentiate them.

The algorithms and settings without detailed description remain basically the same as those in the original CBM-CFS3 model, see Kurz et al. [10].

With the customisation of the CBM-CFS3, the official model software no longer met our requirements. Therefore, we reprogrammed the model in light of customisation and the remaining unchanged algorithms based on Java and R languages, and then applied it to simulate the forest C budgets in Guizhou, China for the period 1990–2016.

### Model input and parameterisation

#### Stand volume growth models

We used the equations developed by Tang et al. [41] to estimate the stand volume and AGB from the FRPDSGP data provided as the model input. The authors developed growth models of stand volume for all the dominant tree species in Guizhou based on the fourth FRPDSGP data, which fully considered the environmental effect on stand volume and applied several approaches of growth function, space-for-time substitution, and zonal-hierarchical method for modelling [41]. A total of 959 growth equations of stand volume were fitted effectively with a five-level stand classifier (i.e. dominant tree species, climatic zone, site quality degree, stand origin, and rocky desertification type).

#### Land use and land cover grids

LULC grids with a 100 m spatial resolution of Guizhou for 1990, 1995, 2000, 2005, 2010, and 2015 were obtained from the continuously updated LULC dataset by the research group of Liu et al. [59–61], which were derived from multi-source high-resolution remote-sensing images and realized using the human–computer interactive interpretation method. The overall accuracies of the six land-use classes, which were the same as the land-use types of FRPDSGP (Table 1), were all above 93%, thus meeting the requirement of user mapping accuracy on a 1:100,000 scale [61].

#### Forest area dynamics

The spatiotemporal dynamics of forest area during 1990–2016 were derived by reconstructing the past forest stand spatial distribution through the fourth FRPDSGP data conducted in 2016 and the LULC grids from 1990 to 2015. The details are described in the Additional file 1: Part II.

#### Disturbance events

Due to the lack of spatiotemporal records of natural disturbances and forest management,^2^ we derived the spatiotemporal distribution of annual disturbance events from 1990 to 2016 from forest area dynamics in accordance with the LUCC rules and our field investigation of forestry construction in Guizhou Province for years. Further, we selected seven major disturbance types (including forest management, hereafter inclusive) associated with LUCC and two major disturbance types associated with forest interior change. The disturbance types, descriptions, and generation rules are listed in Table 2.

**Table 2:** Major disturbance types/forest managements in Guizhou’s forest and their description and generation rules

Disturbance type	Land-use type at last year	Land-use type at current year	Age at last year	Age at current year	Current stand origin	Description	Acronym
Afforestation	Non-forest	Forest land	–	1	Planted	Non–forest land converted to forest land, and the stand was established by afforestation	AF
Natural expansion of forest	Non-forest	Forest land	–	1	Natural	Non-forest land converted to forest land, and the stand was initiated naturally	NE
Deforestation for agriculture	Forest land	Cropland	–	–	–	Forest land converted to cropland after deforestation, which is usually accompanied by salvage, uprooting and burn in Guizhou	DFA
Deforestation for built-up land	Forest land	Built-up land	–	–	–	Forest land converted to built-up land after deforestation thoroughly	DFB
Forest conversion to grassland	Forest land	Grassland	–	–	–	Forest land converted or degraded to grassland owing to human activities or natural causes	FCG
Forest conversion to waters	Forest land	Inland water and wetland	–	–	–	Forest land converted to wetland or reservoirs owing to human activities or natural causes	FCW
Forest degradation to bare land	Forest land	Bare land	–	–	–	Forest land converted or degraded to bare land owing to human activities or natural causes	FDB
Harvest logging	Forest land	Forest land	> = harvest age	–	–	A forest management activity of clear-cut logging to the stands that have reached the harvest age. After that, the land-use type still remains as forest land, whereas the stand age will be reset by plantation or natural expansion of forest; the dominant tree species may be changed	HL
Regeneration logging	Forest land	Forest land	< harvest age	–	–	A forest management activity of regeneration logging to the stands that have not reached the harvest age, in order to restore or enhance the ecological functions of forests. After that, the land-use type still remains as forest land, whereas the stand age will be reset by plantation or natural expansion of forest; the dominant tree species may be changed	RL

The “–” denotes that there is no strict rule for this item

#### Climate data

The climate data included the mean annual temperature and annual precipitation. The historical climate data for 1990–2016 were derived from daily observation data provided by the meteorological stations of Guizhou Province and its surrounding area. The daily observation data were then aggregated yearly and interpolated into 1 km × 1 km grids using thin plate smoothing splines (ANUSPLIN) [62, 63].

#### Parameterisation

The net annual biomass growth increment of each tree species was derived from the related age-to-volume growth curves, volume-to-biomass equations and the multinomial logit model for fitting proportions of biomass (details see Additional file 1: Part III). All the parameters for the estimation of AG and BG biomass C stocks and initial values for BG slow C stocks on non-forest land were collected or calculated from numerous local studies in China [31, 32, 52, 64–68], whereas some parameters for the estimation of DOM C stocks were derived from local researches [52, 69, 70], and some were acquired from relevant foreign studies [10, 71] owing to the absence of local data. The age group division and harvest age of each tree species were set up in compliance with the local forestry management regulations [72]. The city boundaries of Guizhou were adopted as administrative boundaries, and the Guizhou ecological subregions from the *Ecological function regionalization of Guizhou Province* published by the Guizhou government [73] were employed as ecological units. The intersection of the two boundaries generated the spatial units (SPUs), which were applied as the basis of the historic natural disturbance-return interval determination for initialisation and modelling of stand volume growth [41]. The historic natural disturbance regime was assumed to be a stand-replacing fire for DOM pool initialisation, and the last known stand-replacing disturbance was set as clear-cut, according to historic county annals and studies [74, 75]. According to the forest management practices in Guizhou, the disturbance impacts on the forest C budget were mainly estimated using the disturbance matrices associated with Mixedwood Plains from the original CBM-CFS3 model, with a slight modification in the proportion of C transferred between biomass pools. Detailed parameter values, sources, and treatment processes are described in Table S2–S16 in the Additional file 1: Part IV.

## Results

### Model validation

**Fig. 4:**
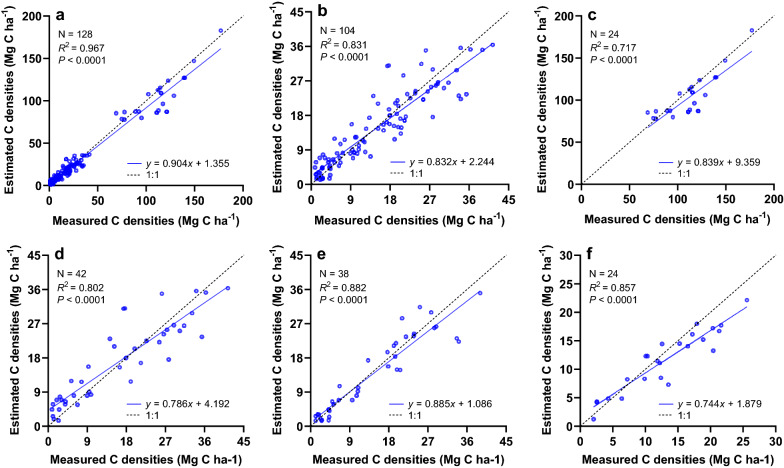
The scatterplot of estimated and measured C densities in different pools and forest types. **a** In all pools and forest types. **b** In biomass, deadwood and litter (BDL) pools. **c** In SOM pool. **d** In BDL pools of coniferous forest type. **e** In BDL pools of broad-leaved forest type. **f** In BDL pools of other forest types, including bamboo forest, economic forest and shrub forest

The results from 1990 to 2016 were verified using a meta-analysis method to examine the performance of our customised model. Substantial C densities (stocks per ha), most measured in situ and some estimated by other methods, in biomass pools and DOM pools of various tree species were collected from numerous relevant studies [e.g. 76–80]. The scatterplot of our estimates and the measured C densities in all tree species and all pools (Fig. 4a) exhibit an adequate linear relationship between the two variables (*R*^2^ = 0.967, *P* < 0.0001); the slope value of the linear fit reached 0.904, almost coinciding with the 1:1 line, indicating a small overall difference between the estimated and measured C densities and the minimal systematic error of our model, which might be related to the accuracy of parameterisation. Furthermore, as the C densities in SOM are much larger than those in other pools, we validated them separately (Fig. 4b, c); in addition, the stimulated biomass and DOM (excluding SOM) C densities were also verified based on different forest types to demonstrate more details (Fig. 4d–f). The slope values of the linear fit ranged from 0.744 to 0.885, and the *R*^2^ values ranged from 0.717 to 0.882, showing the robustness of our model. The validation results imply the high accuracy and reliability of our customised model for estimating the forest C budget in Guizhou.

### Spatiotemporal dynamics of forest C stock

#### Interannual variation of forest area and stand volume

**Fig. 5:**
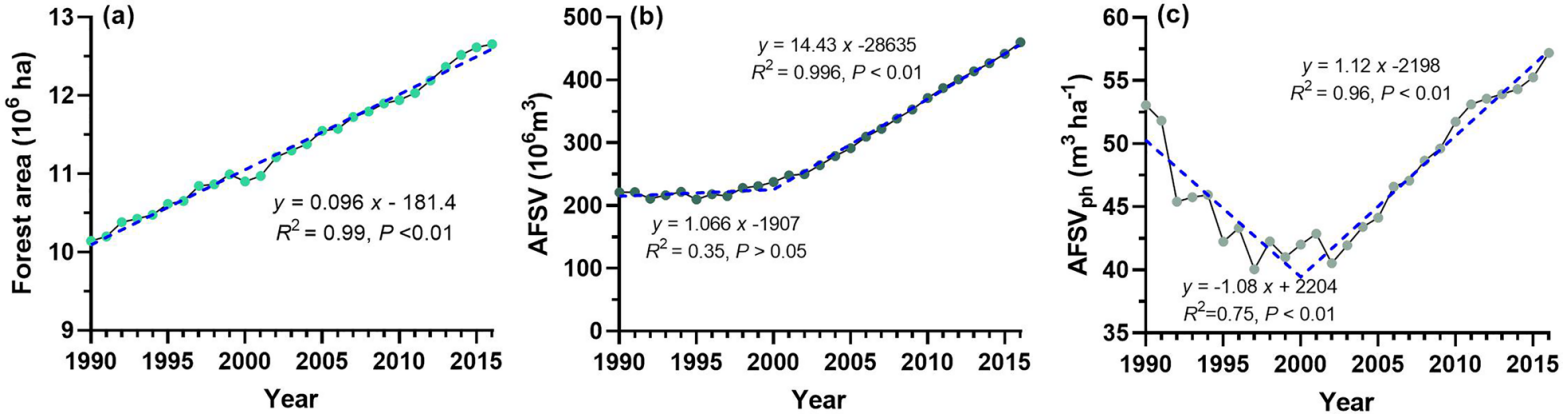
Interannual variation forest area and arbour forest stocking volume from 1990 to 2016. **a** Forest area. **b** Arbour forest stocking volume (AFSV). **c** AFSV per ha (AFSV_ph_)

During 1990–2016, forest areas, including arbour, bamboo, economic, shrub, sparse, and other unclosed forests, in Guizhou had increased steadily and significantly (*P* < 0.01) by an average of 96.24 thousand ha yr^−1^. The forest area in Guizhou reached 12.66 million ha in 2016, an increase of 24.77% from 1990 (Fig. 5a). At the same time, the arbour forest stocking volume^3^ (AFSV) exhibited a fundamentally flat trend in the early period (*slope*^4^ = 1.07, *P* > 0.05) and remarkably increased later (*slope* = 14.43, *P* < 0.01) (Fig. 5b), while the AFSV per ha (AFSV_ph_) decreased rapidly at first (*slope* = − 1.08, *P* < 0.01) but increased significantly afterwards (*slope* = 1.12, *P* < 0.01) (Fig. 5c). The turning points of AFSV and AFSV_ph_ occurred around 2000, which was the first year to implement a series of ecological restoration programs. These findings imply that from 1990 to 2000, the increase in forest area primarily originated from non-arbour forests or young forests, and the area of arbour forests with higher stand volume continued to decline; in other words, overlogging was serious in this period. This situation was controlled after 2000 owing to the implementation of ecological restoration programs.

#### Temporal dynamics of forest C stock

**Fig. 6:**
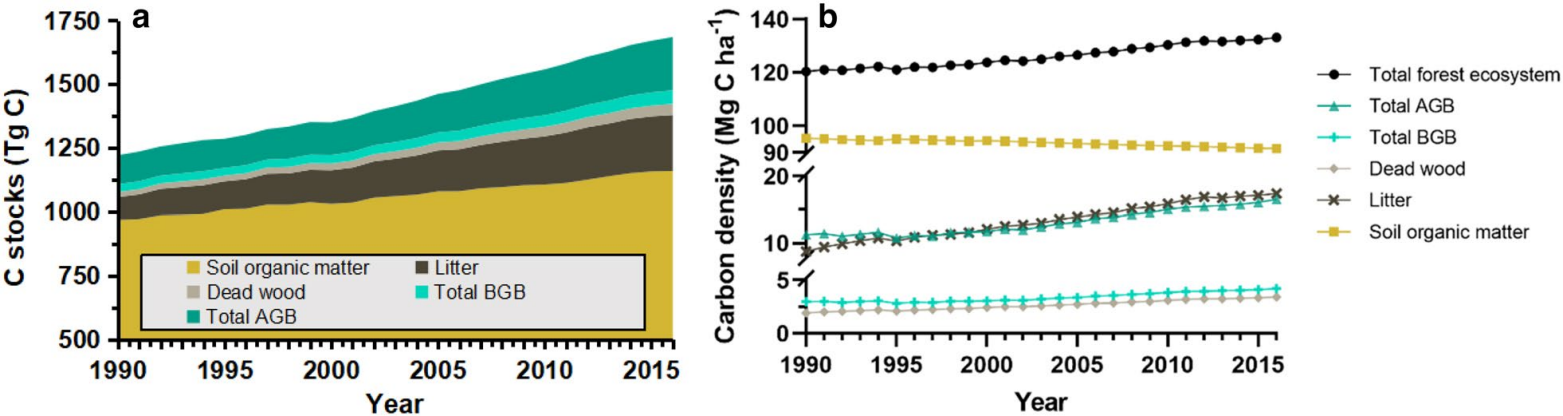
Interannual variation of C stocks and C densities in the GPG pools from 1990 to 2016. **a** C stocks. **b** C densities

Table 3 and Fig. 6 demonstrate the C stock and C density dynamics in the forest ecosystem of Guizhou estimated by the model for the period 1990–2016, including disturbance events. Throughout the simulations, the Guizhou forests were a net carbon sink. In 2016, the total ecosystem C stock reached 1684 Tg C,^5^ increasing from 1220 Tg C in 1990 at a rate of 18 Tg C yr^−1^. Considering the ecosystem C stocks, 15.6% were in the living biomass pools, 68.8% were in the soil, and the remaining 15.6% were in the AG DOM pools. The annual income of ecosystem C density reached 0.53 Mg C ha^−1^, which raised the ecosystem C density from 120 to 133 Mg C ha^−1^, and mostly originated from the litter (67%) and AGB (41%) pools (Table 3). The total ecosystem C stock and C stocks in all five GPG pools increased continuously and significantly during 1990–2016 (*P* < 0.01) (Fig. 6a); such that, the total ecosystem C density and C densities in most GPG pools followed the same trend, except for the soil pool, which continuously decreased during the entire period (*slope*^6^ = − 0.15, *P* < 0.01) (Fig. 6b).

**Table 3:** C stock and C density dynamics in the forest ecosystem of Guizhou for the period 1990–2016, including the disturbance events

	Pool	Living biomass		AG dead organic matter		Soil	Total ecosystem
		AG	BG	Dead wood	Litter		
C stock	1990	114.28	30.16	19.56	89.08	967.12	1220.20
	2016	209.07	53.34	43.32	220.11	1158.35	1684.18
	Change	94.79	23.17	23.76	131.03	191.23	463.98
	Slope	3.86	0.94	0.93	5.12	7.32	18.17
C density	1990	11.27	2.97	1.93	8.78	95.35	120.30
	2016	16.52	4.21	3.42	17.39	91.53	133.08
	Change	5.25	1.24	1.50	8.61	− 3.82	12.78
	Slope	0.23	0.05	0.06	0.34	− 0.15	0.53

In the table, *AG* aboveground, *BG*  belowground

The units of values of 1990, 2016, and changes for C stock are Tg C, whereas those for C density are Mg C ha^−1^

The unit of the slope of C stock is Tg C yr^−1^, whereas that of C density is Mg C ha^−1^ yr^−1^

**Fig. 7:**
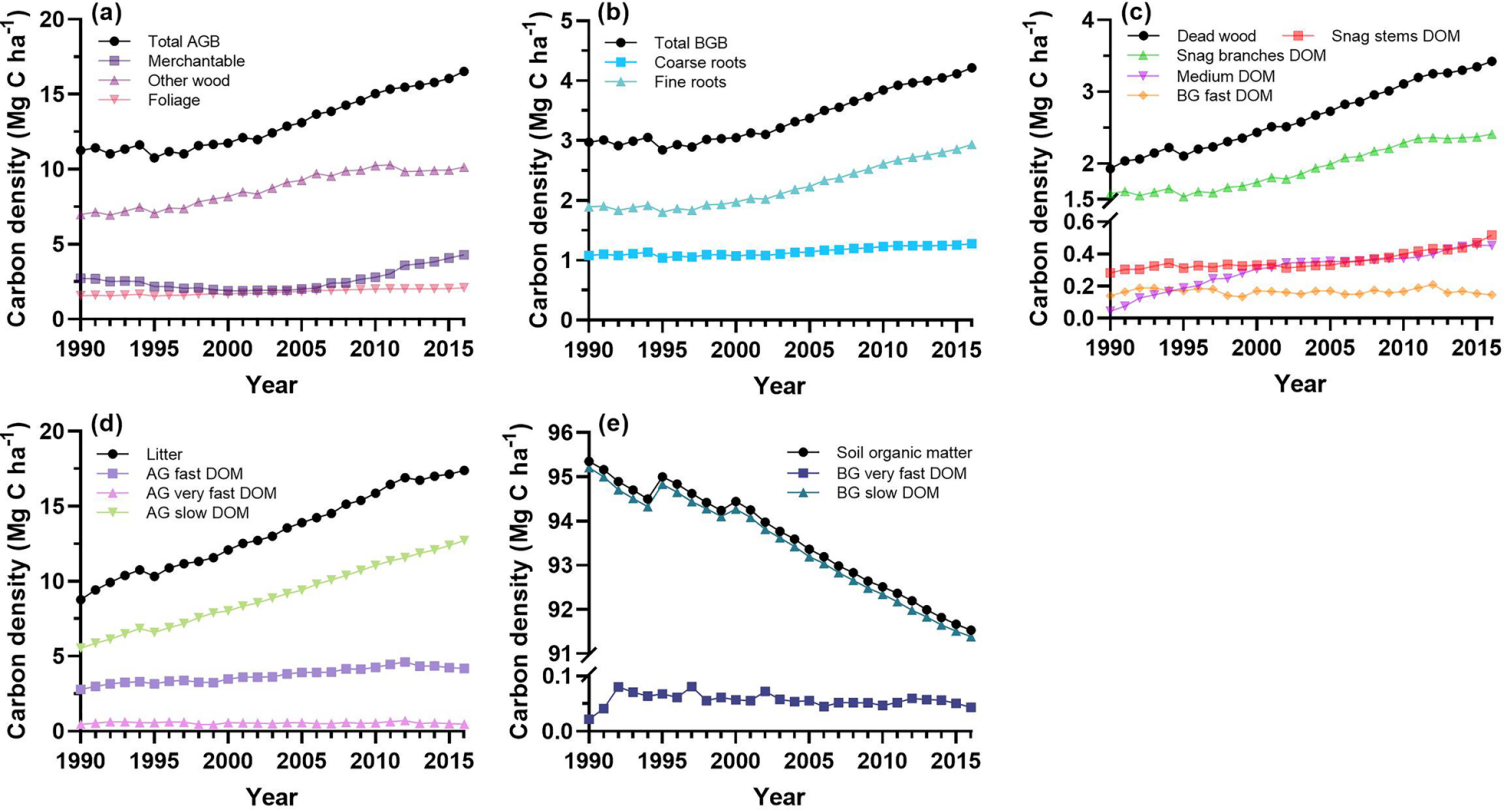
Interannual variation of C densities in the CBM-CFS3 pools from 1990 to 2016. **a** AGB pool. **b** BGB pool. **c** Dead wood pool. **d** Litter pool. **e** Soil organic matter pool

Figure 7 depicts the temporal trends of C densities in all the 14 CBM-CFS3 pools. It is apparent that the C densities in most pools increased significantly in the 26 years: the C density in AG slow DOM pool increased the fastest (*slope* = 0.28, *P* < 0.01), followed by that in other wood (*slope* = 0.15, *P* < 0.01), AG fast DOM (*slope* = 0.06, *P* < 0.01), and merchantable stemwood (*slope* = 0.056, *P* < 0.05); the C densities in BG fast and very fast DOM, AG very fast DOM pools remained essentially stable with minor fluctuations and showed overall slight decreasing tendencies (− 0.0006 to − 0.0001 Mg C ha^−1^ yr^−1^), while the BG slow C density represented a significant downward trend (*slope* = − 0.15, *P* < 0.01).

#### Spatial dynamics of forest C stock

**Fig. 8:**
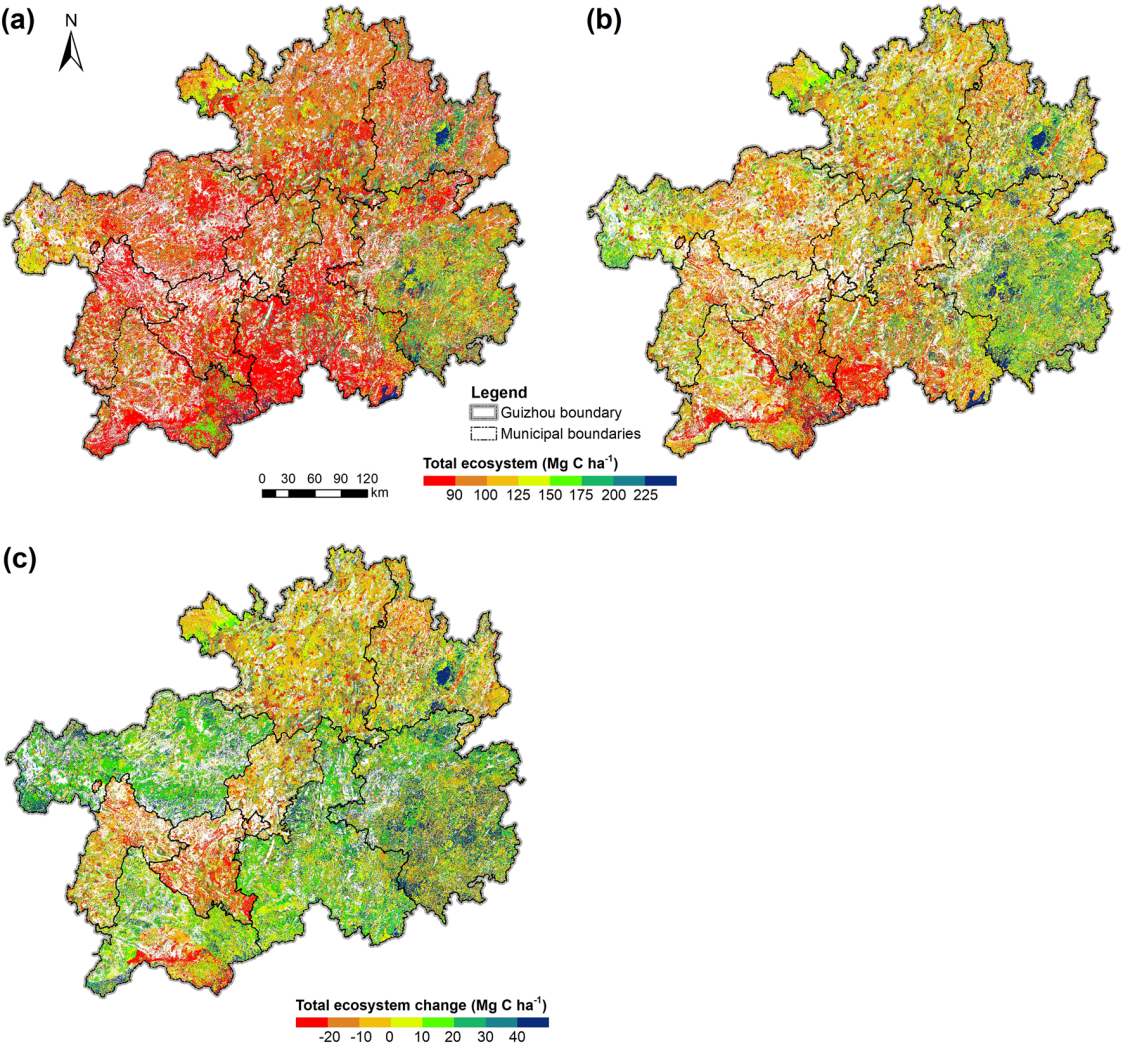
Spatial distributions of total forest ecosystem C density and its change from 1990 to 2016. **a** Ecosystem C density in 1990 and **b** in 2016. **c** Ecosystem C density change from 1990 to 2016

Figure 8 demonstrates the spatial distribution of the total forest ecosystem C stock and its change. There was great spatial heterogeneity in both total forest ecosystem C in 1990 and 2016, and the C densities in the eastern and northern areas were generally higher than those in the western and southern areas (Fig. 8a, b). A large part of the southeast area, that is Qiandongnan City, and the Fanjing Mountain Nature Reserve in Tongren (the bluest patch in the northeast area) were the major C stock areas, while the middle-southern and southwest regions were the opposite. Additionally, the forest C stock was significantly enhanced in most of Guizhou during 1990–2016, with the most remarkable enhancement in the southern and northwestern areas, that is Qiandongnan, Qiannan, Bijie, and north Qianxinan (Fig. 8c), whereby a strong carbon sink function in these areas is depicted. Nevertheless, there were still forest C stock decreases in the northeast and middle-west regions, especially Anshun, Liupanshui, Zunyi, and south Qianxinan, representing themselves as carbon emitter.

**Fig. 9:**
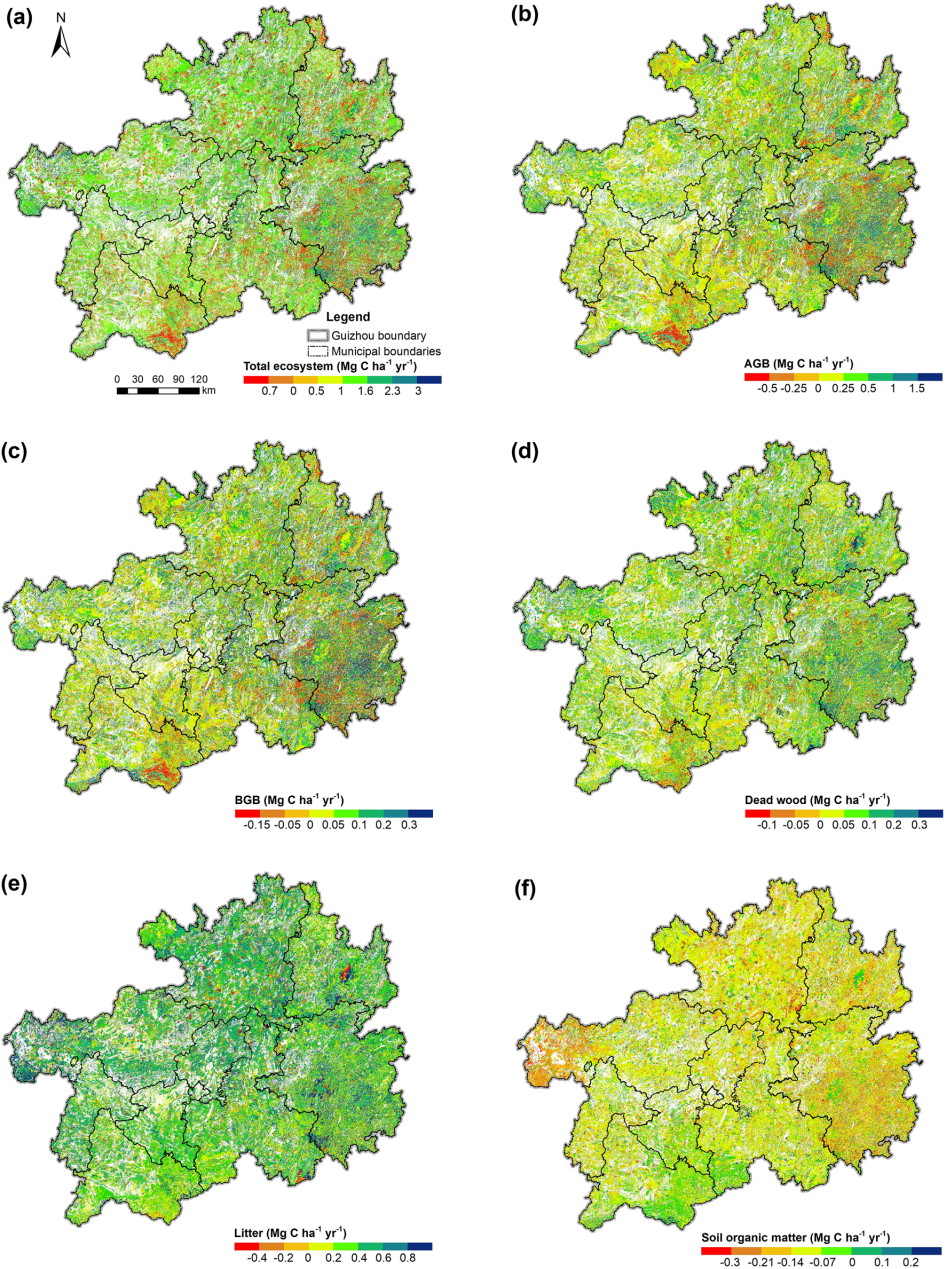
Spatial distribution of slopes of C density changes in the total forest ecosystem and various pools from 1990 to 2016. **a** Total forest ecosystem. **b** AGB pool. **c** BGB pool. **d** Dead wood pool. **e** Litter pool. **f** Soil organic matter pool

As shown in Fig. 9a, the spatial distribution of the slope of total ecosystem C density change is essentially consistent with that of annual C density, higher in the east and north areas and lower in the west and south areas, and the total C density change in most areas reached over 0.5 Mg C ha^−1^ yr^−1^. The slopes of C density changes in various pools followed the same pattern as the total ecosystem, except for the litter and soil pools (Fig. 9b–f). The slope of litter C density change in the north region was slightly higher than that in the south region; the slope of soil C density change, however, showed an overall decreasing trend spatially with a faster decline in the southeast and northwest and a slower decline in the southwest, generally contrary to the slope of the total C density change.

### Spatiotemporal dynamics of disturbance impacts on forest C stock

#### Temporal dynamics of disturbed forest area and converted area

**Fig. 10:**
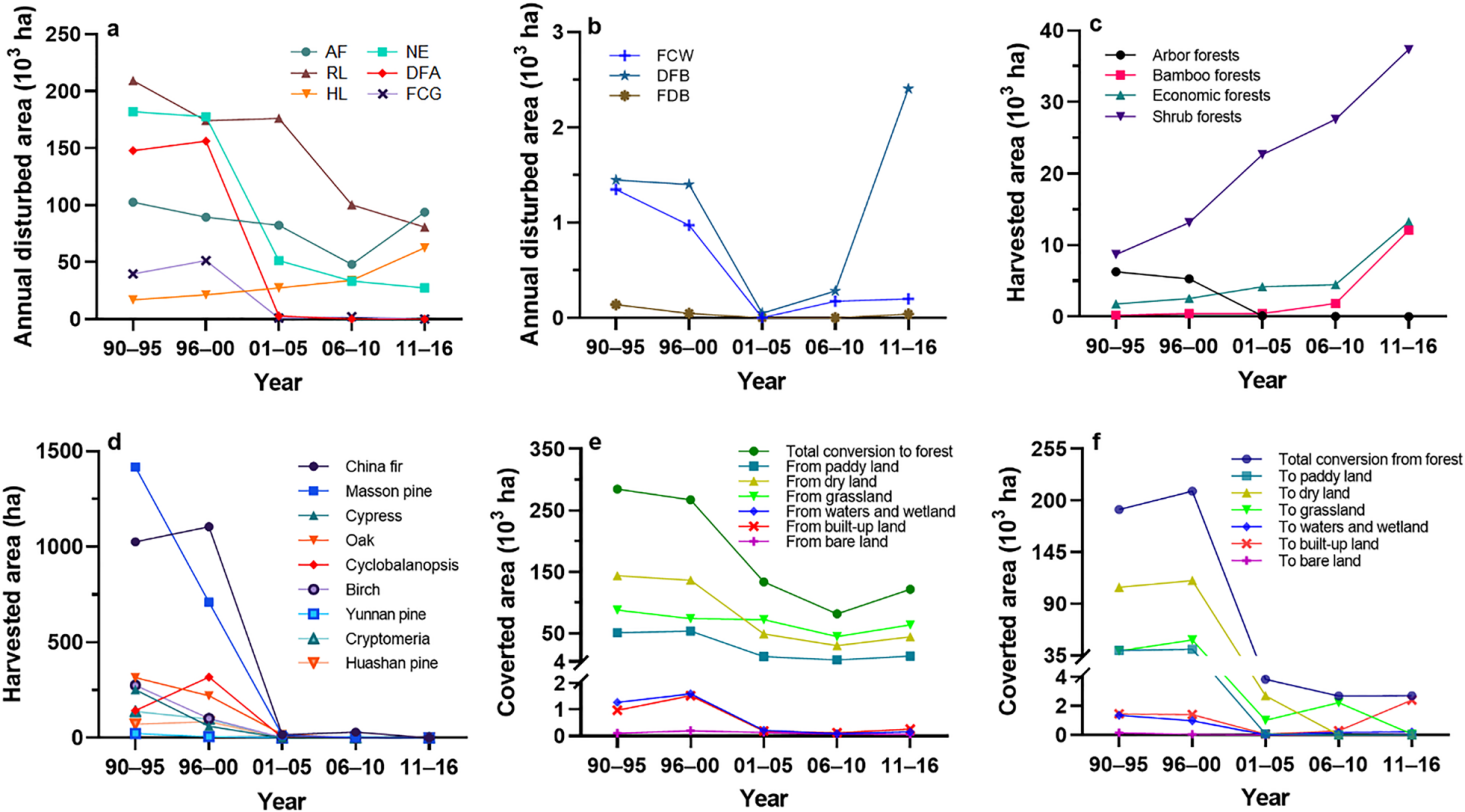
Temporal dynamics of annual disturbed areas, annual harvested areas and annual converted areas. **a** and **b** are the annual disturbed area dynamics of various disturbances types. **c** and **d** are the annual harvested area dynamics of various forest types and major tree species, respectively. **e** and **f** are the dynamics of annual converted area from various non-forest land-use types to forest and from forest to various non-forest land-use types, respectively. In the figure **a** and **b**, AF = afforestation, NE = natural expansion of forest, RL = regeneration logging, HL = harvest logging, DFA = deforestation for agriculture, FCG = forest conversion to grassland, FCW = forest conversion to water, DFB = deforestation for built-up land, FDB = forest degradation to bare land. The annual values are the mean values of every five years, in order to be consistent with the time gap of the contiguous land use and land cover grids

From 1990 to 2016, the total disturbed forest area reached 11.27 million ha, occupying 3.8% of the forest area annually (Table 4). The regeneration logging (RL) was the dominant disturbance type, its annual disturbed area accounted for 33.6% of all disturbed area, followed by those of afforestation (AF) (21.2%) and natural expansion of forest (NE) (19.3%). Figure 10a–b illustrates the temporal dynamics of the annual disturbed area. It is clear that most disturbances, including RL, NE, deforestation for agriculture (DFA), forest conversion to grassland (FCG), forest conversion to water (FCW), and forest degradation to bare land (FDB), declined after 2000. In contrast, the area of harvest logging (HL) continued to rise over the entire period, and the areas of AF and deforestation for built-up land (DFB) showed a downward trend at first, but an upward trend later. By further examining the harvest area, as illustrated in Fig. 10c–d, we determined that the increasing harvest area originated from non-arbour forests, that is, bamboo forest, economic forest, and shrub forest, and HL in major tree species dropped dramatically after 2000. As shown in Fig. 10e–f, almost all the annual converted areas of various LUCC types associated with forest showed decreasing trends after 2000, but the decline of conversion from forest was larger than the decline of conversion to forest overall, thus leading to a net increase in forest area, which increased with minor fluctuations. The interconversion between cropland and forest and between grassland and forest played primary roles in forest land conversion.

**Table 4:** Total disturbed area, total forest C stock changes and C expenditures resulting from disturbances from 1990 to 2016

Disturbance type	Total disturbed area (10^4^ ha)	C stock changes (Gg C)			C expenditures (Gg C)		
		Biomass	DOM	Total	Atmosphere	Products	Total
AF	217.59	6423.51	147.71	6571.23	644.50	0	644.50
NE	238.63	3129.35	− 323.82	2805.53	417.82	0	417.82
RL	378.28	− 77,615.25	− 3153.29	− 80,768.54	42,755.87	47,537.96	90,293.82
HL	87.46	− 9738.20	− 931.75	− 10,669.95	6541.89	4393.88	10,935.77
DFA	153.40	− 14,632.83	560.67	− 14,072.16	10,757.85	5547.02	16,304.87
FCG	47.10	− 4913.52	3760.49	− 1153.03	410.85	1456.66	1867.51
FCW	1.37	− 104.01	114.65	10.64	1.47	5.88	7.35
DFB	3.03	− 260.97	− 66.54	− 327.51	82.33	295.60	377.93
FDB	0.12	− 24.68	14.62	− 10.06	2.59	9.75	12.34
Total	1126.97	− 97,736.61	122.76	− 97,613.86	61,615.16	59,246.74	120,861.90

#### Temporal dynamics of forest C stock change and C expenditure caused by disturbances

The C stock change caused by disturbances refers to the forest C stock increases (e.g. afforestation) or decreases (e.g. deforestation) resulting from disturbances, while the C expenditure caused by disturbances focuses on C stock removal from the forest ecosystem owing to disturbances, including the release to the atmosphere and transfers to the forest products sector. From 1990 to 2016, as reported in Table 4, the ecosystem C stock decrease caused by disturbances reached 97.6 Tg C in total and 3.75 Tg C per year, tantamount to 21% of the total ecosystem C stock change; Table 4 also revealed that the total ecosystem C decrease is primarily the consequence of biomass C loss, as the DOM C increased slightly owing to the transfers from biomass C. The C expenditure caused by disturbances reached 120.9 Tg C in total and 4.65 Tg C per year, of which 51% was released as fluxes to the atmosphere, and the rest were transferred to forest products. RL caused the largest C stock decrease and C expenditure (accounting for 75% of the total C expenditure), followed by DFA (13%) and HL (9%); AF and NE contributed the largest C stock increases (Table 4).

**Fig. 11:**
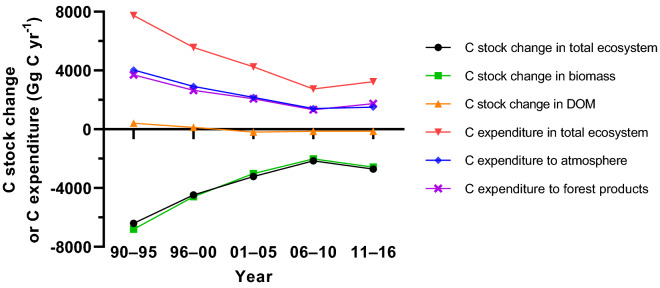
Temporal dynamics of annual total C stock changes and C expenditures resulting from all the disturbances

As illustrated in Fig. 11, both the total C stock changes in the total ecosystem and biomass caused by disturbances were represented as C stock decreases (C change < 0), but both the decrements descended in general with slopes of − 214.40 Gg C yr^−1^ (*P* < 0.01) and − 188.42 Gg C yr^−1^ (*P* < 0.01), respectively; the DOM C change represented a slight increase at first (C change > 0) and then a slight decrease after 2000 (C change < 0), with an overall declining tendency (*slope*^7^ = − 25.99, *P* < 0.01). Overall, the C expenditure in total ecosystem (*slope* = − 230.21, *P* < 0.01), release to the atmosphere (*slope* = − 128.13, *P* < 0.01), and transfers to the forest products (*slope* = − 102.08, *P* < 0.01) caused by disturbances reduced yearly.

**Fig. 12:**
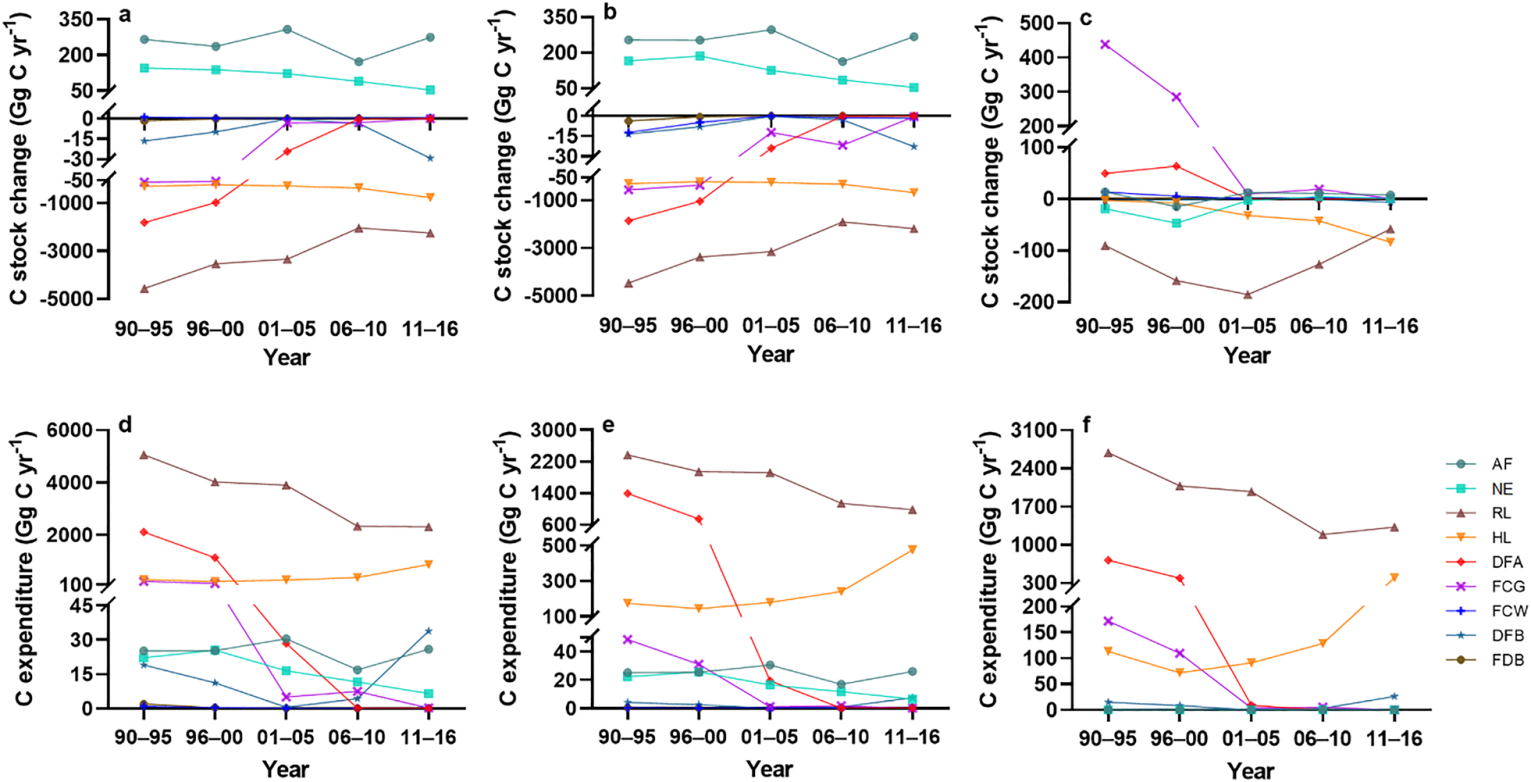
Temporal dynamics of annual forest C stock changes and C expenditures resulting from different disturbances. **a**–**c** are the annual C stock change dynamics resulting from various disturbances types in total ecosystem, biomass and DOM pool, respectively. **d**–**f** are the annual C expenditures resulting from various disturbances types in total ecosystem, to atmosphere and to forest products, respectively

The temporal dynamics of the annual C stock changes and C expenditures resulting from different disturbances are shown in Fig. 12. What stands out in this figure is the significant decreasing trends in C stock change and C expenditure caused by most disturbances, especially DFA, RL, and FCG. The fastest decline in biomass C decrease contributed to the fastest decline in ecosystem C decrease and C expenditure in RL, which respectively reached − 119.18 Gg C yr^−1^ (*P* < 0.01) and − 140.76 Gg C yr^−1^ (*P* < 0.01), while the sharp drop in biomass C decrease and DOM C increase both led to the steep fall of ecosystem C decreases in DFA (*slope* = − 89.53, *P* < 0.01) and FCG (*slope* = − 6.85, *P* < 0.01), so were the ecosystem C expenditure changes they caused. In contrast, there was a slight increase in the ecosystem C decrease caused by HL (*slope* = 21.31, *P* < 0.01) and DFB (*slope* = 21.31, *P* < 0.01), as well as the ecosystem C expenditures caused by HL (*slope* = 26.19, *P* < 0.01) and DFB (*slope* = 0.48, *P* < 0.01). Surprisingly, the ecosystem C increase caused by AF showed an overall declining trend (*slope* = − 0.87, *P* < 0.05), contrary to its annual area change, which might be related to the afforested tree species. In each disturbance, the annual changes in C released to the atmosphere and C transferred to forest products followed the same patterns.

#### Spatial dynamics of forest C stock change and C expenditure caused by disturbances

**Fig. 13:**
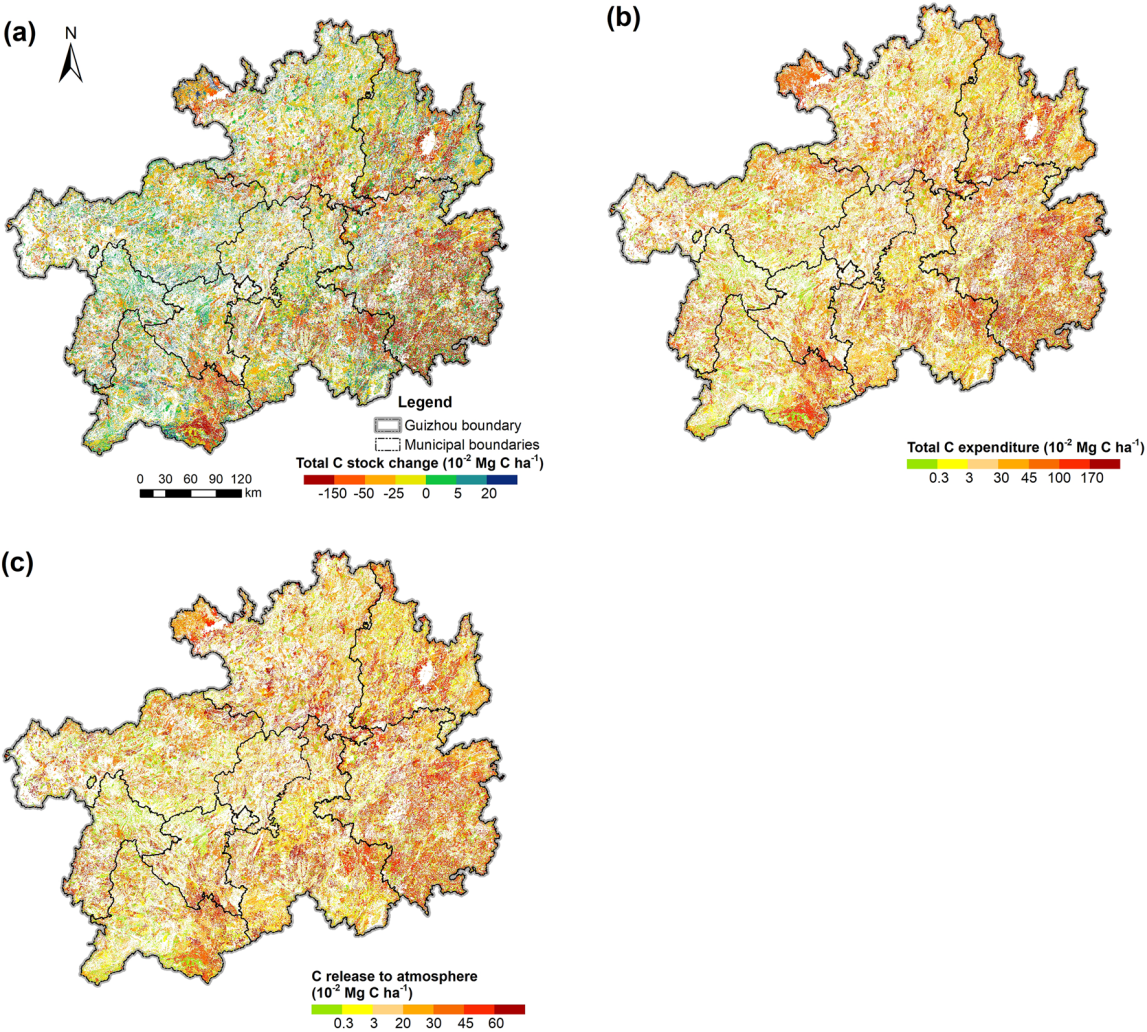
Spatial distribution of total ecosystem C stock change, C expenditure and C released to atmosphere resulting from disturbances from 1990 to 2016. **a** C stock change and **b** C expenditure in total ecosystem. **c** C released to atmosphere resulting from disturbances

Figure 13 demonstrates the spatial dynamics of the total C stock change and C expenditure caused by disturbances during 1990–2016. The ecosystem C stock increases, low ecosystem C expenditure, and C released to the atmosphere resulting from disturbances were mainly distributed in the western and northern areas, whereas the C stock decrease and high C expenditure by disturbances were remarkably concentrated in the eastern and southern regions, especially Qiandongnan, middle Qiannan, and southeastern Qianxinan; similar trends were noticed in northwestern Zunyi and middle Tongren (except the Fanjing Mountain Nature Reserve). Compared with Fig. 8, this figure indicates that the historical negative disturbances (which cause C stock decrease and high C expenditure, e.g. RL, DFA, HL, etc.) that occurred in the southwest regions contributed to its relatively low ecosystem C stock, and the high ecosystem C stock in the southeast areas magnified the disturbance impacts therein. GHG emissions were comparably high in the eastern and southern regions.

## Discussion and suggestions

### Discussion

As illustrated in Fig. 7, merchantable stemwood C density in Guizhou had undergone three change stages: first decreasing in 1990–2000, then holding steadily in 2000–2005, and increasing rapidly after 2005, while the other wood C density continued to grow over the entire period. This is mainly because the C stock in other wood pool mostly originate from immature arbour forests, economic forests, and shrub forests, whereas the C stock in the merchantable stemwood pool primarily originate from mature and post-mature arbour forests. Before 2000, the merchantable stemwood C continued to decrease owing to the overlogging, at the same time, the increase of economic forests and shrub forests improved the C in other wood pool. As afforestation and forest restoration were conducted largely in 2000, the other wood C increased rapidly immediately, while the increase in merchantable stemwood C lagged behind owing to the time for tree growth and maturity; therefore, the merchantable stemwood C density remained steady between 2000 and 2005, and then grew quickly after 2005, with a growth rate much higher than that of other wood C. It can be foreseen that with the maintenance of current forest conservation practice, the merchantable stemwood C density will exceed the other wood C density in the future. In addition, the relatively high temperature in Guizhou resulted in high temperature-dependent decay rates of DOM pools, thus the C stocks that remained in the snag, medium, fast, and very fast DOM pools were considerably low while the slow C remaining in soil were comparably large owing to the quick decomposition and consequent transfer of organic matter; another reason why the C stocks in snag stem and medium pools were small is that there were few stemwood residuals left on the ground as most merchantable stemwood was transferred to the forest products sector after harvest in Guizhou.

The variation in the trend of DOM C density may be related to the deforestation history and climate change in Guizhou Province. From the 1950s to the 1980s, forests in Guizhou suffered serious deforestation, whereby forest coverage decreased from 45% to 12.6% [41, 74]. Consequently, there were massive wood residues which input large C stocks to DOM pools, especially the BG slow DOM pool with the slowest decay rate. During the study period, the temperature in Guizhou increased significantly overall (see Additional file 1: Part V), leading to a remarkable increase in the temperature-dependent decay rates of the DOM pools. As the afforestation period in Guizhou is relatively short, the DOM inputs in some dead wood and litter DOM pools, such as the BG fast DOM and AG very fast DOM pools, obtained from the newly grown forest were less than the DOM decomposed, resulting in decreases in C stocks. In terms of SOM pools, including the BG very fast DOM and BG slow DOM pools, the C stock changes in response to climatic warming depend on how C inputs to soil by NPP and C outputs by SOM decomposition are balanced relative to each other [81–83]. Climatic warming not only promotes photosynthesis and plant growth but also accelerates the respiration and decomposition of SOM, thus having both positive [84–86] and negative effects [87–89] on SOM C stock. During the study period, the SOM C output, that is, the amount of C in the massive SOM obtained from the historical deforestation, which was decayed and released to the atmosphere, increased owing to continuous warming, while the SOM C input from the short-term afforestation was comparably small, thus leading to the consecutive decline of SOM C. In the future, when the newly added forest grows to maturity, the annual SOM C input could probably be higher than the SOM C output, whereby the decreasing trend of C stocks in the relevant SOM pools would be reversed. Notwithstanding, the impact of climatic warming on future SOM C stock changes in Guizhou should be thoroughly studied before making assertion.

GHGs are considered the main driver of global warming, and human activity is regarded as the primary contributor to GHG emissionsc [90–92]. The CBM-CFS3 reports GHG fluxes associated with disturbances and land-use changes [10]. In this study, AF, NE, FCG, FCW, and FDB only emit CO_2_ because there is no burning, whereas RL, HL, DFA, and DFB result in both CO_2_ and non-CO_2_ emissions (i.e. CH_4_, CO, and N_2_O) from burned organic matter. Accordingly, during 1990–2016, a total of 204 million tonnes of CO_2_, 802 thousand tonnes of CH_4_, 12.6 million tonnes of CO, and 34 thousand tonnes of N_2_O were emitted by disturbances in the study area, totalling 231 million CO_2_ equivalents (CO_2_e).^8^ The largest GHG emissions were observed in RL (69.43% of the total), followed by DFA (17.47%), and HL (10.62%); the total GHG emissions of the remaining disturbance types were less than 3%. It should be noted that our model only accounts for the disturbance impact on C stock and C expenditure in the year the disturbance occurred, and does not involve post-disturbance dynamics.

### Implications and suggestions

According to our study, the expansion of forest area and increase in forest age are both essential for the improvement of forest C stocks and carbon sink capacity. However, forest expansion area becomes limited as the total land stock becomes inadequate; therefore, more attention should be paid to forest conservation to enhance forest age and quality. Previous studies have revealed that forest age plays a significant role in influencing forest production, decomposition, and net carbon accumulation [9, 93]. Our results also indicated that despite the increase in forest area, the rapid growth of C stocks in litter and AGB pools, which were brought on by arbour forest growth, contributed greatly to the forest stock increase in Guizhou. Therefore, priority should be given to the protection and restoration of existing old-growth forests, cultivation of young planted forests, and management of artificial forests, apart from afforestation and reforestation [94]. The establishment of nature reserves, such as the Fanjing Mountain Nature Reserve, is a practical approach for implementing these measures.

In addition, it is also helpful to improve the forest C sequestration capacity by afforesting local tree species with high C sequestration potential, in accordance with the local climate and geographical characteristics. According to our study, the total ecosystem C densities of arbour species were 2–135% higher than those of bamboo species, 25–190% higher than those of economic and shrub species, and the total ecosystem C densities of most broad-leaved species were higher than those of coniferous species (Additional file 1: Table S15). Beech (*Fagus* spp.), phoebe (*Phoebe* spp.), maple (*Acer* spp.), Katus (*Castanopsis* spp.), cyclobalanopsis, Chinese yew (*Taxus wallichiana* var. chinensis [Pilger] Florin), and Huashan pine (*Pinus armandii* Franch.) were the local tree species with the highest C sequestration potential. Hitherto, most forest areas in Guizhou have been covered by shrub forests with low C sequestration owing to karst rocky desertification, which implies the high C sequestration potential of forests in Guizhou, and thus urges the local government to take concrete measures to improve the soil and water conservation capacity.

Nevertheless, the impacts of human activities and climate change on forest C stocks should not be overlooked. Most disturbances cause C expenditures and GHG emissions to the atmosphere, exacerbating global warming; meanwhile, climate change, especially climatic warming, leads to DOM C stock loss and CO_2_ release, further intensifying warming. The local forestry department can reduce the negative impacts of anthropogenic disturbances on forest C stock as much as possible, such as extending thinning intervals rationally, minimising burning during logging activities, and reducing forest conversion, especially deforestation.

### Uncertainties

Although we estimated C stock values and levels of change within forests in Guizhou, including all the GPG and CBM-CFS3 pools and disturbance impacts, and verified the results, uncertainties remain. In this study, a large amount of measured data were collected from substantial studies of local, neighbouring areas, or other similar climatic regions in China for parameterisation as realistically as possible; however, because some parameters are unique to the CBM-CFS3 model and there are fewer empirical studies in China, we adopted the default model parameter values and other measured data from Europe, such as the disturbance matrices and base decay rates, which may have given rise to unforeseen errors. In addition, as the FRPDS only records the stand age and stand volume per hectare of stands with DBH ≥ 5 cm, there might be errors in estimating the stand volumes in early growth by our age-to-volume growth models for the lack of data at this growth stage in the model fitting. In future studies, field experiments will be conducted in Guizhou to collect local measurements for all key parameters and stand volumes, thereby enhancing the accuracy and reliability of our model.

Compared with other terrestrial ecosystems, the C cycle in karst areas is more special and complex because of the reversible chemical processes of dissolution, transfer, and deposition of carbonate rocks [95]. Although we considered the particularity of karst area in the biomass C estimation by using the rocky desertification type as a stand classifier in the stand volume growth modelling, the specificity and complexity of the C cycle in the karst soil pool were still absent, which should be addressed in future studies.

In addition, we did not consider other natural (wildfire, disease and insect pests, wind damage, snow damage, landslide) and anthropogenic (tending thinning, fertilisation, illegal activities) disturbances that occur frequently in Guizhou. This is attributed to the absence of detailed disturbance spatiotemporal records. Moreover, the CBM-CFS3 model only considers the temperature effect on C stock change at present, while some important influences on the C cycle, such as atmospheric CO_2_ fertilisation [96], nitrogen deposition [97], precipitation [98], and solar radiation [99], still lack consideration. We expect that, with the development of remote sensing technology and organic coupling with biophysiological process models, these studies can be further advanced.

## Conclusions

The main goal of this study was to customise the CBM-CFS3 model for forest C budget estimation in China and further apply the customised model to Guizhou, which is of great importance to the national forest C sink capacity in China, to examine its adaptation and accuracy. The customisation involved the modification of the AGB C stock algorithm, addition of a carbon budget accounting for bamboo forests, economic forests, and shrub forests, improvement of non-forest land BG slow DOM pool initialisation, and other model settings, according to the local forest inventory system, forest type, and ecological environment in Guizhou. The adequate linear relationship between the estimated and measured C densities (*R*^2^ = 0.967, *P* < 0.0001, *slope* = 0.904) in the model validation demonstrated the high accuracy and reliability of our customised model.

The simulation of spatiotemporal dynamics of forest C stocks and disturbance impacts in Guizhou for the period 1990–2016 was conducted based on the customised model. The results showed that Guizhou forests were a net carbon sink under large-scale afforestation throughout the study period; the total ecosystem C stock and C density, and C stocks in all five GPG pools increased continuously and significantly, while the soil C density decreased over the entire period, which might be attributed to the deforestation history and climate change. The total ecosystem C stock increased from 1220 Tg C in 1990 to 1684 Tg C in 2016 at a rate of 18 Tg C yr^−1^, which was significantly enhanced in most areas, with the most remarkable values in the southern and northwestern areas. The total ecosystem C stock decrease and C expenditure caused by disturbances reached 97.6 Tg C and 120.9 Tg C, respectively, but both represented significant decreasing trends owing to the decline of disturbed forest area during 1990–2016; RL, DFA and HL caused the largest C stock decrease and C expenditure, and AF and NE contributed the largest C stock increases.

In conclusion, our study demonstrated that, after effective customisation, the CBM-CFS3 can be successfully applied to simulate forest C dynamics in Guizhou. This study provides the foundation for the application of CBM-CFS3 in China and further enlightens model customisation in other areas. Further studies will focus on the reduction of uncertainty, including the improvement of parameterisation, addition of more disturbance types, influences of karst landforms, and atmospheric and climatic effects on the C cycle.

## Supplementary Information

**Additional file 1**: **Part I.** Detailed information about the main tree species in Guizhou.** Table S1.** Forest type, dominant tree species (group) (DTSG), and their cover areas in Guizhou in 2016. **Part II.** Reconstruction of the past forest stand spatial distribution during 1990–2016. **Part III.** Assumptions for net annual biomass growth increment. **Part IV.** Detailed parameters for forest carbon (C) stocks estimation in Guizhou using customised CBM-CFS3 model. **Table S2. **Volume-to-stemwood biomass model parameters by DTSG. **Table S3.** Multinomial logit model parameters of for fitting proportions of biomass in stemwood, bark, branches and foliage to total tree biomass by DTSG. **Table S4.** Bamboo biomass per unit area in stemwood, branches, foliage, coarse roots and fine roots at different age. **Table S5.** Economic forest and shrub forest biomass per unit area in stemwood, other wood, foliage and roots. **Table S6.** C content values by dominant tree species. **Table S7.** Decomposition parameters to simulate DOM dynamics. **Table S8.** Turnover rates of stemwood, other wood, foliage and roots biomass by forest types. **Table S9.** Default initial values for belowground slow C density on non-forest land (0-100 cm). **Table S10.** Age group division for DTSG in general arbour forest. **Table S11.** Age group division for DTSG in short-rotation plantation, and fast-growing/high-yield plantation. **Table S12.** Age group division for bamboo species. **Table S13.** Harvest age of DTSG in public welfare forest. **Table S14.** Harvest age of DTSG in timber plantation. **Table S15.** Annual C stock and C density of each DTSG in Guizhou from 1990 to 2016. **Table S16. **Disturbance matrices in Guizhou. **Fig. S1.** Guizhou spatial unit distribution. **Part V.** Annual temperature trend during 1990–2016 in Guizhou.

## Data Availability

The datasets during and/or analyzed during the current study available from the corresponding author on reasonable request.

## Abbreviations

C: Carbon
CBM-CFS3: Carbon Budget Model of the Canadian Forest Sector
CO_2_: Carbon dioxide
GHG: Greenhouse gas
DOM: Dead organic matter
LULC: Land use and land cover
LUCC: Land use and land cover changes
IPCC: Intergovernmental Panel on Climate Change
GPG: Good Practice Guidance
AG: Aboveground
BG: Belowground
SOM: Soil organic matter
AGB: Aboveground biomass
BGB: Belowground biomass
FRPDS: Forest Resource Planning and Design Survey
FRPDSGP: Forest Resource Planning and Design Survey in Guizhou Province
DBH: Diameter at breast height
MST: Merchantable-sized trees
NST: Non-merchantable-sized trees
SST: Sapling-sized trees
YF: Young forests
MAF: Middle-aged forests
NMF: Near-mature forests
MF: Mature forests
PMF: Post-mature forests
biomass_pua_: Biomass per unit area
UNFCCC: United Nations Framework Convention on Climate Change
SPUs: Spatial units
R^2^: Determination coefficient
AFSV: Arbour forest stocking volume
AFSV_ph_: Arbour forest stocking volume per ha
AF: Afforestation
NE: Natural expansion of forest
DFA: Deforestation for agriculture
DFB: Deforestation for built-up land
FCG: Forest conversion to grassland
FCW: Forest conversion to waters
FDB: Forest degradation to bare land
HL: Harvest logging
RL: Regeneration logging
CO_2_e: CO_2_ equivalents

## Footnotes

1 According to the UNFCCC, the length of time that land remains in a conversion category after a change in land use is by default 20 years. After 20 years, the area converted enters the remaining category, and no further DOM changes are assumed.

2 The natural disturbances and forest management data is confidential and mastered by local government.

3 Only the stand volumes of arbour forest were recorded in the FRPDSGP and estimated in the model.

4 The unit of the slope of annual AFSV change is 10^6^ m^3^ yr^−1^, whereas that of annual AFSV_ph_ change is m^3^ ha^−1^ yr^−1^.

5 1 Tg C = 10^6^ tonnes of C; 1 Gg C = 10^3^ tonnes of C; 1 Mg C = 1 tonnes of C.

6 The unit of the slope of annual C stock change is Tg C yr^−1^, whereas that of annual C density change is Mg C ha^−1^ yr^−1^.

7 The units of the slope of annual C stock change and C expenditure caused by disturbances are both Gg C yr^−1^.

8 The “global warming potential (GWP) of GHGs in a centennial time frame” is adopted according to the *Guidelines for Compiling Greenhouse Gas Inventories in Guizhou province (trial)*. GWP: CH_4_ 21; N_2_O 310.
