# Supplementary material for: Spatiotemporal dynamics of forest ecosystem carbon budget in Guizhou: customisation and application of the CBM-CFS3 model for China
Source: Carbon Balance Manag. 2022 Jul 2;17:10. doi: 10.1186/s13021-022-00210-0 (PMC9250733; doi:10.1186/s13021-022-00210-0)
Supplement: Supplementary file 1 — Additional file 1: Part I. Detailed information about the main tree species in Guizhou. Table S1. Forest type, dominant tree species (group) (DTSG), and their cover areas in Guizhou in 2016. Part II. Reconstruction of the past forest stand spatial distribution during 1990–2016. Part III. Assumptions for net annual biomass growth increment. Part IV. Detailed parameters for forest carbon (C) stocks estimation in Guizhou using customised CBM-CFS3 model. Table S2. Volume-to-stemwood biomass model parameters by DTSG. Table S3. Multinomial logit model parameters of for fitting proportions of biomass in stemwood, bark, branches and foliage to total tree biomass by DTSG. Table S4. Bamboo biomass per unit area in stemwood, branches, foliage, coarse roots and fine roots at different age. Table S5. Economic forest and shrub forest biomass per unit area in stemwood, other wood, foliage and roots. Table S6. C content values by dominant tree species. Table S7. Decomposition parameters to simulate DOM dynamics. Table S8. Turnover rates of stemwood, other wood, foliage and roots biomass by forest types. Table S9. Default initial values for belowground slow C density on non-forest land (0-100 cm). Table S10. Age group division for DTSG in general arbour forest. Table S11. Age group division for DTSG in short-rotation plantation, and fast-growing/high-yield plantation. Table S12. Age group division for bamboo species. Table S13. Harvest age of DTSG in public welfare forest. Table S14. Harvest age of DTSG in timber plantation. Table S15. Annual C stock and C density of each DTSG in Guizhou from 1990 to 2016. Table S16. Disturbance matrices in Guizhou. Fig. S1. Guizhou spatial unit distribution. Part V. Annual temperature trend during 1990–2016 in Guizhou. [file 13021_2022_210_MOESM1_ESM.docx]

**Part I**

Detailed information about the main tree species in Guizhou

**Table S1** Forest type, dominant tree species (group) (DTSG), and their cover areas in Guizhou in 2016. DCF = deciduous coniferous forest, ECF = evergreen coniferous forest, DBF = deciduous broad-leaved forest, EBF = evergreen broad-leaved forest, MF = mixed forest, N/A = not applicable. The proportion values are accurate to 2 decimal places if they are greater than 0.01%, otherwise are accurate to 3 decimal places. The table did not include the sparse and unclosed forests.

| No. | Forest type | DTSG | | Area  (10^3^ ha) | Proportion (%) | Vegetation type |
| --- | --- | --- | --- | --- | --- | --- |
|  |  | Common name | Scientific name |  |  |  |
| 1 | Coniferous  forests | Fir | *Abies fabri* (Mast.) Craib | 0.52 | 0.005 | DCF |
| 2 |  | Spruce | *Picea asperata* Mast. | 0.63 | 0.006 | DCF |
| 3 |  | Chinese yew | *Taxus wallichiana* var. chinensis (Pilger) Florin | 3.86 | 0.04 | ECF |
| 4 |  | Masson pine | *Pinus massoniana* Lamb. | 1684.39 | 15.65 | ECF |
| 5 |  | Huashan pine | *Pinus armandii* Franch. | 157.06 | 1.46 | ECF |
| 6 |  | Yunnan pine | *Pinus yunnanensis* Franch. | 173.62 | 1.61 | ECF |
| 7 |  | Other pine | *Pinus* spp. | 15.49 | 0.14 | ECF |
| 8 |  | Keteleeria | *Keteleeria fortunei* (Murr.) Carr. | 3.77 | 0.04 | ECF |
| 9 |  | Hemlock | *Tsuga chinensis* (Franch.) Pritz. | 0.09 | 0.001 | ECF |
| 10 |  | Cypress | *Cupressus funebris* Endl. | 323.77 | 3.01 | ECF |
| 11 |  | Other cypress | *Cupressus* spp. | 40.18 | 0.37 | ECF |
| 12 |  | Chinese fir | *Cunninghamia lanceolata* (Lamb.) Hook. | 1695.29 | 15.75 | ECF |
| 13 |  | Cryptomeria | *Cryptomeria japonica* var. sinensis | 168.91 | 1.57 | ECF |
| 14 |  | Metasequoia | *Metasequoia glyptostroboides* Hu & W. C. Cheng | 1.74 | 0.02 | ECF |
| 15 |  | Other fir | *Cunninghamia* spp. | 1.34 | 0.01 | MF |
| 16 |  | Other coniferous tree species (groups) | N/A | 1.98 | 0.02 | MF |
| 17 | Broad-leaved forests | Poplar/aspen | *Populus* spp. | 80.10 | 0.74 | DBF |
| 18 |  | Willow | *Salix* spp. | 0.82 | 0.008 | DBF |
| 19 |  | Eucalyptus | *Eucalyptus* spp. | 45.00 | 0.42 | EBF |
| 20 |  | Camphor | *Cinnamomum* spp. | 9.62 | 0.09 | EBF |
| 21 |  | Phoebe | *Phoebe* spp. | 3.10 | 0.03 | EBF |
| 22 |  | Oak | *Quercus* spp. | 220.50 | 2.05 | MF |
| 23 |  | Cyclobalanopsis | *Cyclobalanopsis* spp. | 212.38 | 1.97 | EBF |
| 24 |  | Beech | *Fagus* spp. | 2.89 | 0.03 | DBF |
| 25 |  | Birch | *Betula* spp. | 177.97 | 1.65 | DBF |
| 26 |  | Basswood | *Tilia tuan* Szyszyl. | 0.21 | 0.002 | DBF |
| 27 |  | Locust | *Robinia pseudoacacia* L. | 18.78 | 0.17 | DBF |
| 28 |  | Katus | *Castanopsis* spp. | 17.34 | 0.16 | EBF |
| 29 |  | Maple | *Acer* spp. | 1.69 | 0.02 | DBF |
| 30 |  | Melia | *Melia azedarach* L. | 3.21 | 0.03 | DBF |
| 31 |  | Chinese toon | *Toona sinensis* (A. Juss.) Roem. | 21.27 | 0.20 | DBF |
| 32 |  | Elm | *Ulmus* spp. | 8.70 | 0.08 | DBF |
| 33 |  | Ebony | *Dalbergia* spp. | 0.78 | 0.007 | DBF |
| 34 |  | Firmiana | *Firmiana* spp. | 5.36 | 0.05 | DBF |
| 35 |  | Other broad-leaved tree species (groups) | N/A | 1666.48 | 15.53 | MF |
| 36 |  | Broad-leaved mixed tree species (groups) | N/A | 474.21 | 4.41 | MF |
| 37 | Bamboo forests | Moso bamboo | *Phyllostachys heterocycla cv. Pubescens* | 67.31 | 0.63 | EBF |
| 38 |  | Phyllostachys | Phyllostachys spp. | 6.22 | 0.06 | EBF |
| 39 |  | Dendrocalamus | Dendrocalamus spp. | 24.02 | 0.22 | EBF |
| 40 |  | Chimonobambusa | Chimonobambusa spp. | 10.59 | 0.10 | EBF |
| 41 |  | Bambusa | Bambusa spp. | 19.60 | 0.18 | EBF |
| 42 |  | Other bamboo species | N/A | 37.30 | 0.35 | MF |
| 43 | Economic forests | Walnut | *Juglans regia* | 137.83 | 1.28 | DBF |
| 44 |  | Prunus | *Prunus* spp. | 94.15 | 0.87 | DBF |
| 45 |  | Other fruit tree species | N/A | 156.85 | 1.46 | MF |
| 46 |  | Oil-tea | *Camellia* oleifera | 62.48 | 0.58 | EBF |
| 47 |  | Other edible oil tree species | N/A | 0.30 | 0.00 | MF |
| 48 |  | Tea | *Camellia* spp. | 95.71 | 0.89 | EBF |
| 49 |  | Flavorings tree species | N/A | 9.23 | 0.09 | MF |
| 50 |  | Medicinal materials tree species | N/A | 48.84 | 0.45 | MF |
| 51 |  | Industrial raw materials tree species | N/A | 37.38 | 0.35 | MF |
| 52 |  | Other economic tree species | N/A | 1.64 | 0.02 | MF |
| 53 | Shrub forests | Oak shrub | *Quercus* spp. | 387.55 | 3.60 | DBF |
| 54 |  | Firethorn | *Pyracantha fortuneana* | 156.67 | 1.46 | EBF |
| 55 |  | Itea | *Itea* spp. | 40.50 | 0.38 | MF |
| 56 |  | Azalea | *Rhododendron* spp. | 20.35 | 0.19 | EBF |
| 57 |  | Bamboo shrub | N/A | 31.37 | 0.29 | EBF |
| 58 |  | Other shrub species | N/A | 2072.99 | 19.26 | MF |
| 59 | Total | N/A | N/A | 10761.93 | 100.00 | N/A |

**Part II**

Reconstruction of the past forest stand spatial distribution during 1990–2016

To learn the spatio-temporal dynamics of forest area during 1990–2016, we reconstructed the past forest stand spatial distribution through the fourth FRPDSGP data conducted in 2016 and the LULC grids from 1990 to 2015. The age of each stand in the FRPDSGP was decreased by 26 years (backwards from 2016 to 1990) and the corresponding volume was recalculated by applying the related stand volume growth model and new age. A present stand age ≤ 26 years indicates that the stand is newly established after 1990 and the established year can be obtained by subtracting the age from 2016. For the land parcels before forest stand established or has no age recorded (including economic forest land, shrub forest land and non-forest land), we obtained their land-use types in 1990, 1995, 2000, 2005, 2010 and 2015 by overlaying the six LULC grids on the FRPDSGP data. The land-use type of each land parcel remained unchanged in the 4-years gap between two LULC grids because the gap is comparatively short, and LUCC is highly correlated with the government’s Five-Year Plan in China, which is consistent with the LULC year (e.g., 2001–2005). For those land parcels with a land-use type of forest (FLPs), their tree species were assumed to be the same as the most common tree species of surroundings, and their ages from 1991 to 2016 were set to be 1-year-old at the first year they emerged. As for the FLPs’ age in the start year of 1990, since relevant researches had revealed that the Guizhou’s forest coverage had dropped to 12.6% in the early 1980s due to large-scale deforestation for reclamation and steelmaking (Pu et al., 1988; Xu, 2010), considering the substantial stands with present age > 26 years, we assumed the FLPs existed in 1990 were newly established after 1980 and set their ages in 1990 to the values less or equal to 10, depending on the counties where they grows and the counties historical forestry materials. The ages of FLPs were then increased year by year.

**Part III**

Assumptions for net annual biomass growth increment

The net annual growth increment of stemwood biomass is derived from the age-to-volume growth curves and volume-to-biomass equations, which can be expressed as follow:

ΔBio = (*f*(age, classifiers) - *f*(age-1, classifiers)) **g*(a, b, classifiers)

*ΔBio_stem_* = (*Vol*(*age*, classifiers) -*Vol*(*age*-1, classifiers)) **B*(*a*, *b*, classifiers)

where *ΔBio_stem_* is the net growth increment of stemwood biomass; *Vol*(*age*, classifiers) is the corresponding age-to-volume growth curve; *B*(*a*, *b*, classifiers) is the corresponding volume-to-biomass equation; *age* is the stand age in the target year, *age*-1 is the stand age in the last year; classifiers mean the combination of relevant classifiers, which are five classifiers (DTSG, climatic zone, site quality degree, stand origin, rocky desertification type) in the age-to-volume growth curves (see Tang et al. (2021)), and one classifier (DTSG) in the volume-to-biomass equations (Table S2); *a*, *b* are parameters in the volume-to-biomass equations (Table S2).

The net growth increment of bark, branches or foliage biomass can be calculated by the stemwood biomass in the target year and in the last year and the corresponding multinomial logit model for fitting proportions of biomass:

*ΔBio_bk_bh_fg_* = *Bio_stem_*(*age*)/*P_stem_*(*Vol*(*age*))**P**_bk_bh_fg_*(*Vol*(*age*))

- *Bio_stem_*(*age*-1)/*P_stem_*(*Vol*(*age*-1))**P_bk_bh_fg_*(*Vol*(*age*-1))

*Bio_stem_*(*age*) = *Vol*(*age*, classifiers)* *B*(*a*, *b*, classifiers)

where *ΔBio_bk_bh_fg_* is the net growth increment of bark, branches or foliage biomass; *Bio_stem_*(*age*) is the stemwood biomass in the target year; *P_stem_*(*Vol*(*age*)) is the corresponding proportion equation of stemwood biomass to total tree biomass (Table S3); *P_bk_bh_fg_* (*Vol*(*age*)) is the corresponding proportion equation of bark, branches or foliage biomass to total tree biomass (Table S3).

**Part IV**

Detailed parameters for forest carbon (C) stocks estimation in Guizhou using customised CBM-CFS3 model^^[[1]](#footnote-1)^^

**Table S2** Volume-to-stemwood biomass (*B_stem_*) model parameters by DTSG.

*B_stem_* = *a* × *volume^b^*

| DTSG | *a* | *b* | *R*^2^ | Study area | Reference |
| --- | --- | --- | --- | --- | --- |
| Masson pine | 0.583 | 0.943 | 0.890 | Hunan, Sichuan, Anhui, Fujian, Guizhou, Guangxi | Fu (2013) |
| Chinese fir | 0.256 | 1.027 | 0.931 | Hunan, Jiangxi, Guizhou, Guangxi, Fujian |  |
| Cypress | 0.968 | 0.804 | 0.802 | Sichuan, Hunan, Hubei, Fujian |  |
| Huashan pine | 1.175 | 0.717 | 0.575 | Shaanxi, Gansu, Ningxia, Yunnan |  |
| Elm, ebony, basswood, melia | 0.45 | 1.045 | 0.984 | Anhui, Hunan, Hubei, Gansu, Shaanxi |  |
| Broad-leaved mixed tree species (groups), Other broad-leaved tree species (groups) | 2.411 | 0.66 | 0.984 | Sichuan, Hunan, Fujian, Guangxi |  |
| Other coniferous tree species (groups) | 0.405 | 1 | 1 | Hunan, Sichuan, Gansu, Fujian |  |
| Fir, spruce, Chinese yew, other fir | 0.2993 | 1.016 | 0.9697 | Yunnan | Feng (2014) |
| Yunnan pine, keteleeria, other pine | 0.2596 | 1.0244 | 0.9499 | Yunnan |  |
| Oak, katus | 0.4825 | 1.038 | 0.9033 | Anhui, Fujian, Guangdong, Guangxi, Guizhou, Hainan, Henan, Hunan, Jiangxi, Sichuan, Shaanxi, Beijing |  |
| Camphor, Chinese toon, maple | 1.0415 | 0.8663 | 0.6604 | Beijing, Hunan, Jiangsu, Shaanxi |  |
| Cryptomeria, metasequoia | 0.4978 | 0.9195 | 0.8942 | Fujian, Guangxi, Guangdong, Henan, Hunan, Jiangsu, Shandong, Sichuan |  |
| Locust | 1.140 | 0.804 | 0.915 | Sichuan, Anhui, Hubei, Beijing, Hebei, Shanxi, Henan, Shaanxi | Luo et al. (2013) |
| Poplar, willow, birch | 1.180 | 0.881 | 0.807 | Fujian, Yunnan, Shandong, Jiangsu, Shanxi, Xizang, Inner Mongolia |  |
| Firmiana, beech | 1.135 | 0.870 | 0.888 | Fujian, Hunan, Yunnan, Hubei |  |
| Phoebe, cyclobalanopsis | 1.079 | 0.817 | 0.828 | Fujian, Sichuan, Hunan, Yunnan, Anhui, Zhejiang |  |
| Eucalyptus | 1.167 | 0.652 | 0.868 | Fujian, Guangdong, Yunnan, Sichuan, Hunan, Guangxi, Hainan |  |

**Table S3** Multinomial logit model parameters of for fitting proportions of biomass in stemwood (*P_stem_*), bark (*P_bark_*), branches (*P_branch_*) and foliage (*P_foliage_*) to total tree biomass by DTSG. “-” in the table indicates that the value is not listed in the original references.

$$P_{stem}=\frac{1}{1+e^{a1+a2\times vol+a3\times lvol}+e^{b1+b2\times vol+b3\times lvol}+e^{c1+c2\times vol+c3\times lvol}}$$

$$P_{bark}=\frac{e^{a1+a2\times vol+a3\times lvol}}{1+e^{a1+a2\times vol+a3\times lvol}+e^{b1+b2\times vol+b3\times lvol}+e^{c1+c2\times vol+c3\times lvol}}$$

$$P_{branch}=\frac{e^{b1+b2\times vol+b3\times lvol}}{1+e^{a1+a2\times vol+a3\times lvol}+e^{b1+b2\times vol+b3\times lvol}+e^{c1+c2\times vol+c3\times lvol}}$$

$$P_{foliage}=\frac{e^{c1+c2\times vol+c3\times lvol}}{1+e^{a1+a2\times vol+a3\times lvol}+e^{b1+b2\times vol+b3\times lvol}+e^{c1+c2\times vol+c3\times lvol}}$$

| DTSG | *a*1 | *a*2 | *a*3 | *b*1 | *b*2 | *b*3 | *c*1 | *c*2 | *c*3 | *R*^2^ | | | | Reference |
| --- | --- | --- | --- | --- | --- | --- | --- | --- | --- | --- | --- | --- | --- | --- |
|  |  |  |  |  |  |  |  |  |  | Stem | Bark | Branches | Foliage |  |
| Masson pine | -0.6068 | 0.0010 | -0.3250 | -0.0556 | -0.0006 | -0.2754 | 1.3574 | 0.0021 | -0.8236 | 0.506 | 0.038 | 0.290 | 0.633 | Fu (2013) |
| Chinese fir | -1.6652 | -0.0005 | -0.0013 | 0.7408 | -0.0015 | -0.4148 | 1.3848 | -0.0031 | -0.4774 | 0.809 | 0.308 | 0.679 | 0.818 |  |
| Cypress | -2.0669 | -0.0013 | 0.0873 | -0.5136 | -0.0014 | -0.1200 | -1.3521 | -0.0098 | 0.2299 | 0.586 | 0.101 | 0.208 | 0.538 |  |
| Huashan pine | 0.3935 | 0.0042 | -0.5830 | 5.6418 | 0.0093 | -1.5249 | 6.9700 | 0.0183 | -2.2488 | 0.670 | 0.263 | 0.709 | 0.203 |  |
| Poplar, willow, birch, firmiana, beech | -0.6395 | 0.0005 | -0.2023 | 0.2622 | 0.0007 | -0.3224 | 0.3162 | -0.0031 | -0.5788 | 0.757 | 0.019 | 0.334 | 0.763 |  |
| Phoebe, Locust, Cyclobalanopsis | -0.9106 | 0.0023 | -0.3015 | -0.1500 | -0.0039 | -0.1706 | -3.6862 | -0.0464 | 1.0922 | 0.585 | 0.132 | 0.184 | 0.134 |  |
| Broad-leaved mixed tree species (groups), Other broad-leaved tree species (groups) | 1.5547 | 0.0039 | -0.8643 | 0.5795 | -0.0014 | -0.3127 | 2.8135 | 0.0037 | -1.0861 | 0.314 | 0.477 | 0.180 | 0.106 |  |
| Other coniferous tree species (groups) | -2.6462 | -0.0031 | 0.2743 | -9.1546 | -0.0255 | 2.3370 | -2.2178 | -0.0039 | 0.2278 | 0.638 | 0.112 | 0.759 | 0.025 |  |
| Fir, spruce, Chinese yew, other fir | -1.7653 | -0.0009 | 0.0025 | 0.1013 | 0.0005 | -0.2874 | -0.3062 | -0.0067 | -0.2181 | - | 0.650 | 0.249 | 0.448 | Feng (2014) |
| Yunnan pine, keteleeria, other pine | -0.4717 | 0.0005 | -0.2318 | 0.3374 | 0.0003 | -0.3357 | 0.5552 | -0.0020 | -0.6635 | - | 0.792 | 0.207 | 0.561 |  |
| Oak, katus | -1.6773 | -0.0003 | -0.0179 | -0.7054 | -0.0009 | -0.1388 | -0.6225 | -0.0038 | -0.0805 | - | 0.050 | 0.197 | 0.400 |  |
| Eucalyptus | 0.5182 | -0.0020 | -0.2762 | -0.0115 | -0.0043 | -0.0268 | 0.4148 | -0.0101 | -0.2336 | - | 0.626 | 0.708 | 0.770 |  |
| Camphor, Chinese toon, maple | -0.4192 | -0.0012 | -0.2088 | 0.7682 | -0.0014 | -0.2470 | 0.2703 | -0.0059 | -0.1646 | - | 0.081 | 0.273 | 0.480 |  |

**Table S4** Bamboo biomass per unit area in stemwood, branches, foliage, coarse roots and fine roots at different age (unit: t ha^-1^). The data were estimated from GPDRC (2017), the fourth FRPDSGP and Wu (1983).

| Age | Biomass pool | | | Tree proportion | | | | |
| --- | --- | --- | --- | --- | --- | --- | --- | --- |
|  | AG | BG | Total | Stemwood | Branches | Foliage | Coarse roots | Fine roots |
| 1 | 20.40 | 5.54 | 25.94 | 16.01 | 3.25 | 1.14 | 2.14 | 3.40 |
| 2 | 33.65 | 9.14 | 42.80 | 27.44 | 3.47 | 2.74 | 3.53 | 5.61 |
| 3 | 33.88 | 9.21 | 43.08 | 27.63 | 3.56 | 2.70 | 3.56 | 5.65 |
| 4 | 34.10 | 9.27 | 43.37 | 27.80 | 3.65 | 2.65 | 3.58 | 5.69 |
| 5 | 34.36 | 9.34 | 43.70 | 28.00 | 3.68 | 2.68 | 3.61 | 5.73 |
| 6 | 34.62 | 9.41 | 44.03 | 28.20 | 3.72 | 2.70 | 3.63 | 5.78 |
| 7 | 34.13 | 9.27 | 43.41 | 28.24 | 3.45 | 2.44 | 3.58 | 5.69 |
| 8 | 33.65 | 9.14 | 42.79 | 28.28 | 3.18 | 2.19 | 3.53 | 5.61 |
| 9 | 33.56 | 9.12 | 42.68 | 28.17 | 3.18 | 2.21 | 3.52 | 5.60 |
| 10+* | 33.47 | 9.10 | 42.57 | 28.05 | 3.17 | 2.25 | 3.51 | 5.59 |

*Note：According to Wu (1983), the physiological mechanisms of an individual Moso bamboo, the most representative bamboo species covering more than two-thirds bamboo forest area, start to decline when it reaches above 8 years old, and its productivity gradually declines and consumption dominates, and gradually aging and dying over 10 years in general. Therefore, we assumed the biomass per unit area of bamboo remained unchanged after reaching 10 years of age.

**Table S5** Economic forest and shrub forest biomass per unit area in stemwood, other wood, foliage and roots (unit: t ha^-1^). The data were estimated from GPDRC (2017), the fourth FRPDSGP and studies of Wen et al. (2015) and Li (2017).

| Tree proportion | Economic forest | Shrub forest |
| --- | --- | --- |
| Stemwood | 10.32 | 0 |
| Other wood | 5.12 | 10.34 |
| Foliage | 2.36 | 2.2 |
| Roots | 3.28 | 3.2 |

**Table S6** C content values by dominant tree species (unit: %)

| DTSG | C content | Study area | Reference |
| --- | --- | --- | --- |
| Fir | 0.5079 | Xianggeri, northwest Yunnan | Wang et al. (2012) |
| Spruce | 0.5160 | Yunnan | Tang (2007) |
| Chinese yew | 0.5185 | Hubei | Wang et al. (2016) |
| Masson pine | 0.5984 | Qiannan and Qiannan, Guizhou | Yang (2015) |
| Huashan pine | 0.5437 | Yunnan | Tang (2007) |
| Yunnan pine | 0.5148 | Xianggeri, northwest Yunnan | Wang et al. (2012) |
| Other pine | 0.511 | Nationwide | PRC (2013) |
| Keteleeria | 0.5133 | Yunnan | Hou et al. (2018) |
| Hemlock | 0.502 | Nationwide | PRC (2013) |
| Cypress | 0.5211 | Yunnan | PRC (2013) |
| Other cypress | 0.510 | Nationwide | PRC (2013) |
| Chinese fir | 0.5528 | Qiannan and Qiannan, Guizhou | Yang (2015) |
| Cryptomeria | 0.5479 | Yunnan | Tang (2007) |
| Metasequoia | 0.5489 | Yunnan | Tang (2007) |
| Other fir | 0.5489 | Yunnan | Tang (2007) |
| Other coniferous tree species (groups) | 0.510 | Nationwide | PRC (2013) |
| Poplar/aspen | 0.4956 | Yunnan | Tang (2007) |
| Willow | 0.485 | Nationwide | PRC (2013) |
| Eucalyptus | 0.5019 | Yunnan | Tang (2007) |
| Camphor | 0.4535 | Guiyang, Guizhou | He et al. (2007a) |
| Phoebe | 0.5050 | Yunnan | Tang (2007) |
| Oak | 0.4801 | Qiannan and Qiannan, Guizhou | Yang (2015) |
| Cyclobalanopsis | 0.4938 | Yunnan | Tang (2007) |
| Beech | 0.439 | Nationwide | PRC (2013) |
| Birch | 0.4474 | Guiyang, Guizhou | He et al. (2007a) |
| Basswood | 0.4623 | Pingjiang, Hunan | Shen (2017) |
| Locust | 0.5041 | Yunnan | Tang (2007) |
| Katus | 0.47 | Nationwide | PRC (2013) |
| Maple | 0.4404 | Central and northern Henan | Bai et al. (2017) |
| Melia | 0.4675 | Yunnan | Tang (2007) |
| Chinese toon | 0.4675 | Yunnan | Tang (2007) |
| Elm | 0.497 | Nationwide | PRC (2013) |
| Ebony | 0.497 | Nationwide | PRC (2013) |
| Firmiana | 0.47 | Nationwide | PRC (2013) |
| Other broad-leaved tree species (groups) | 0.490 | Nationwide | PRC (2013) |
| Broad-leaved mixed tree species (groups) | 0.490 | Nationwide | PRC (2013) |
| Bamboo species | 0.4705 | Hubei | He et al. (2007b) |
| Economic species | 0.48 | Nationwide | Wen et al. (2015), Li (2017) |
| Shrub species | 0.47 | Nationwide | Li (2017) |

**Table S7** Decomposition parameters to simulate DOM dynamics. Decomposition parameters include the base decay rate at a reference temperature of 10 ℃, sensitivity to temperature (*Q*_10_), and the proportion of decay C released to the atmosphere (*P_atm_*) versus transferred to a slow DOM pool (*P_t_*), where *P_atm_* + *P_t_* = 1. AG = aboveground, BG = belowground, N/A = not applicable.

| DOM pool | Base decay rate (yr^−1^) | *Q*_10_ | *P_atm_* | *P_t_* | Reference |
| --- | --- | --- | --- | --- | --- |
| AG very fast | 0.4316 | 2.78 | 0.815 | 0.115 | Huang et al. (2010) |
| AG fast | 0.1900 | 3.51 | 0.810 | 0.190 | Smyth et al. (2010) |
| AG slow | 0.0150 | 2.65 | 1 | N/A |  |
| BG fast | 0.2140 | 4.19 | 0.810 | 0.190 |  |
| BG very fast | 0.4030 | 2.95 | 0.830 | 0.170 |  |
| BG slow | 0.0032 | 0.90 | 1 | N/A |  |

**Table S8** Turnover rates of stemwood, other wood, foliage and roots biomass by forest types (unit: a^-1^)

| Vegetation type | Stemwood | Other wood | Foliage | Roots | Study area | Reference |
| --- | --- | --- | --- | --- | --- | --- |
| EBF | 0.44 | 0.0311 | 0.6135 | 0.0345 | Stemwood came from Guizhou, other wood, foliage and roots from nationwide | GPDRC (2017)  Zhou et al. (2010) |
| DBF |  | 0.0289 | 0.9524 | 0.0272 |  |  |
| ECF |  | 0.0194 | 0.3356 | 0.0178 |  |  |
| DCF |  | 0.0177 | 0.8850 | 0.0173 |  |  |
| MF |  | 0.0261 | 0.5882 | 0.0230 |  |  |

**Table S9** Default initial values for belowground slow C density on non-forest land (0-100 cm) (unit: Mg C ha^-1^). The values of inland water and built-up land are set to 0 as there are no vegetation grows. LULC = land use and land cover.

| Vegetation type + LULC classification | Default initial values for belowground slow C density | Reference |
| --- | --- | --- |
| Paddy | 118.3 | Huang et al. (2017) |
| Dryland | 89.9 |  |
| Grassland | 80.7 |  |
| Bare land | 71.2 |  |

**Table S10** Age group division for DTSG in general arbour forest. Data are sourced from Guizhou Forestry Bureau (2015), in which the age group division of the same DTSG is different among the stands of general arbour forest, short-rotation plantation, and fast-growing/high-yield plantation. YF = young forest, MAF = middle-aged forest, NMF = near-mature forest, MF = mature forest, PMF = post-mature forest, ACP = Age class period. The age group division of DTSG not mentioned in the table is assigned to that of DTSG with similar bioecological characteristics and main uses, same as below.

| DTSG | Origin | Age group | | | | | ACP |
| --- | --- | --- | --- | --- | --- | --- | --- |
|  |  | YF | MAF | NMF | MF | PMF |  |
| Cypress, hemlock, fir, keteleeria | Natural | ≤ 40 | 41–60 | 61–80 | 81–120 | ≥ 121 | 20 |
|  | Planted | ≤ 20 | 21–40 | 41–60 | 61–80 | ≥ 81 | 20 |
| Chinese fir, cryptomeria, metasequoia, *Taiwania flousiana* | Planted | ≤ 10 | 11–20 | 21–25 | 26–35 | ≥ 36 | 5 |
| Masson pine, Huashan pine, Yunnan pine, and other pine | Natural | ≤ 20 | 21–30 | 31–40 | 41–60 | ≥ 61 | 10 |
|  | Planted | ≤ 10 | 11–20 | 21–30 | 31–50 | ≥ 51 | 10 |
| Poplar, willow, melia, *Paulownia*, *Sassafras*, and other such kind of soft broad-leaved^*^ species | Planted | ≤ 5 | 6–10 | 11–15 | 16–25 | ≥ 26 | 5 |
| Birch, Chinese toon, eucalyptus, *Camptotheca*, *Pterocarya*, *Catalpa bungei*, *Alnus*, *Catalpa*, *Magnolia*, *Davidia*, *Liquidambar*, and other such kind of soft broad-leaved species | Natural | ≤ 20 | 21–40 | 41–50 | 51–70 | ≥ 71 | 10 |
|  | Planted | ≤ 10 | 11–20 | 21–30 | 31–50 | ≥ 51 | 10 |
| Elm, katus, camphor, phoebe, oak, *Sophora*, *Schima*, cyclobalanopsis, *Xylosma*, *Broussonetia kazinoki*, and other such kind of hard broad-leaved species | Natural | ≤ 40 | 41–60 | 61–80 | 81–120 | ≥ 121 | 20 |
|  | Planted | ≤ 20 | 21–40 | 41–50 | 51–70 | ≥ 71 | 10 |

*Note: A soft broad-leaved species refers to the tree species with hardness in wood cross-section less than 701 kg/cm^2^. Conversely, it is a hard broad-leaved species.

**Table S11** Age group division for DTSG in short-rotation plantation, and fast-growing/high-yield plantation.

| DTSG | Age group | | | | | ACP |
| --- | --- | --- | --- | --- | --- | --- |
|  | YF | MAF | NMF | MF | PMF |  |
| Chinese fir, masson pine, exotic pines* | ≤ 5 | 6–10 | 11–15 | 16–20 | ≥ 21 | 5 |
| Poplar, eucalyptus, and other kind of fast-growing broad-leaved species | ≤ 3 | 4–6 | 7–9 | 10–12 | ≥ 13 | 3 |

*Note: Exotic pines include *Pinus elliottii*, *Pinus taeda* and *Pinus caribaea*, same as below.

**Table S12** Age group division for bamboo species. Data are sourced from Guizhou Forestry Bureau (2015), in which the age group of bamboo forest is divided according to the bamboo degree (each bamboo degree contains 2 years): degree 1, degree 2 to 3 and degree 4 correspond to YF, MAF and MF, PMF, respectively.

| Growth stage | YF | MAF and MF | | PMF |
| --- | --- | --- | --- | --- |
| Bamboo degree | degree 1 | degree 2 | degree 3 | degree 4 |
| Age | < 3 | 3–4 | 5–6 | ≥ 7 |

**Table S13** Harvest age of DTSG in public welfare forest. Data are sourced from Guizhou Forestry Bureau (2009) and National Forestry Administration (2005), in which the harvest age of the same DTSG is different among the stands of public welfare forest and timber plantation. The harvest age of stands of public welfare forest is 1–2 age classes greater than that of general timber plantation in timber plantation.

| DTSG | Origin | Harvest age |
| --- | --- | --- |
| Spruce, hemlock | Natural | 121 |
|  | Planted | 101 |
| Fir | Natural | 121 |
|  | Planted | 61 |
| Masson pine, Yunnan pine, Huashan pine, *Pinus tabuliformis*, *Pinus kesiya*, *Pinus densata* | Natural | 61 |
|  | Planted | 51 |
| Chinese fir, cryptomeria, metasequoia | Planted | 36 |
| Poplar, eucalyptus, melia, *Paulownia*, *Casuarina*, *Pterocarya*, *Sophora*, *Betula platyphylla*, *Populus davidiana* | Planted | 26 |
| Birch, elm, *Schima*, *Liquidambar* | Natural | 71 |
|  | Planted | 51 |
| Oak, katus, basswood, *Fraxinus mandschurica*, *Juglans mandshurica*, *Phellodendron amurense* | Natural | 121 |
|  | Planted | 71 |
| *Phyllostachys* | Planted | 7 |

**Table S14** Harvest age of DTSG in timber plantation.

| Timber plantation type | DTSG | Harvest age |
| --- | --- | --- |
| General timber plantation | Chinese fir, cryptomeria | 26 |
|  | Masson pine, Yunnan pine, Huashan pine, exotic pines | 31 |
|  | Poplar, eucalyptus, *Paulownia* | 16 |
| Industrial raw material plantation | Masson pine, exotic pines | 16 |
|  | Poplar, eucalyptus, *Paulownia* | 10 |

**Table S15** Annual C stock and C density of each DTSG in Guizhou from 1990 to 2016 (unit: Mg C ha^-1^). AGB = Aboveground biomass, BGB = Belowground biomass, SOM = Soil organic matter. Mean and SD respectively denotes the annual mean value and standard deviation of C densities of each DTSG from 1990 to 2016 (SD values mainly reflect the variation in the age dependent C densities).

| DTSG | Annual mean ecosystem C stock (Gg C) | Ecosystem C Density | | AGB C Density | | BGB C Density | | Dead wood C Density | | Litter C Density | | SOM C Density | |
| --- | --- | --- | --- | --- | --- | --- | --- | --- | --- | --- | --- | --- | --- |
|  |  | Mean | SD | Mean | SD | Mean | SD | Mean | SD | Mean | SD | Mean | SD |
| Fir | 61.05 | 137.37 | 24.03 | 16.60 | 6.74 | 3.57 | 1.44 | 2.69 | 1.41 | 16.45 | 8.60 | 98.06 | 13.65 |
| Spruce | 69.29 | 136.51 | 50.76 | 14.80 | 7.65 | 3.18 | 1.64 | 2.28 | 1.57 | 12.58 | 8.12 | 103.67 | 39.64 |
| Chinese yew | 188.52 | 212.87 | 74.47 | 30.23 | 17.89 | 6.56 | 3.89 | 5.48 | 4.04 | 23.22 | 15.62 | 147.39 | 41.97 |
| Masson pine | 239018.39 | 168.78 | 42.51 | 24.55 | 12.34 | 5.30 | 2.73 | 4.69 | 2.56 | 17.95 | 8.63 | 116.29 | 24.95 |
| Huashan pine | 22770.94 | 196.56 | 52.47 | 28.49 | 4.42 | 6.23 | 1.00 | 4.90 | 1.71 | 27.97 | 9.54 | 128.98 | 43.79 |
| Yunnan pine | 23248.81 | 146.99 | 28.38 | 16.73 | 7.24 | 3.60 | 1.57 | 3.04 | 1.57 | 15.74 | 7.50 | 107.87 | 18.67 |
| Other pine | 1259.84 | 133.22 | 32.55 | 18.54 | 12.83 | 4.05 | 2.82 | 3.63 | 2.60 | 14.23 | 8.95 | 92.76 | 13.20 |
| Keteleeria | 498.20 | 143.70 | 28.74 | 17.18 | 7.41 | 3.66 | 1.58 | 3.33 | 1.86 | 18.58 | 9.27 | 100.94 | 15.11 |
| Hemlock | 10.82 | 147.86 | 29.57 | 14.86 | 6.72 | 3.22 | 1.50 | 2.70 | 1.80 | 18.31 | 9.64 | 108.77 | 18.96 |
| Cypress | 31097.13 | 134.14 | 27.83 | 16.40 | 6.18 | 3.50 | 1.32 | 2.61 | 1.42 | 15.68 | 8.69 | 95.96 | 16.09 |
| Other cypress | 2740.58 | 125.71 | 31.38 | 13.81 | 8.51 | 2.94 | 1.82 | 2.18 | 1.64 | 12.56 | 9.45 | 94.22 | 17.81 |
| Chinese fir | 211524.68 | 154.87 | 36.21 | 20.84 | 13.59 | 4.55 | 3.02 | 3.98 | 2.75 | 15.08 | 7.65 | 110.42 | 19.21 |
| Cryptomeria | 13097.96 | 138.33 | 38.88 | 18.17 | 11.31 | 3.92 | 2.49 | 3.05 | 2.20 | 13.80 | 8.54 | 99.39 | 21.55 |
| Metasequoia | 129.29 | 132.41 | 33.84 | 16.86 | 9.10 | 3.66 | 2.02 | 2.87 | 2.01 | 12.83 | 6.75 | 96.19 | 21.53 |
| Other fir | 166.25 | 149.86 | 40.21 | 17.60 | 10.94 | 3.84 | 2.40 | 3.10 | 2.04 | 16.42 | 8.89 | 108.90 | 23.81 |
| Other coniferous tree species (groups) | 221.48 | 135.90 | 62.51 | 12.78 | 14.80 | 2.71 | 3.20 | 2.35 | 3.97 | 12.65 | 12.41 | 105.40 | 40.19 |
| Poplar/aspen | 6535.95 | 136.01 | 27.35 | 15.89 | 6.23 | 6.35 | 1.78 | 3.96 | 2.66 | 13.27 | 6.11 | 96.53 | 17.09 |
| Willow | 47.94 | 139.29 | 35.06 | 15.64 | 9.61 | 5.93 | 2.90 | 4.09 | 3.42 | 11.66 | 7.23 | 101.96 | 22.61 |
| Eucalyptus | 3697.98 | 175.35 | 40.94 | 27.33 | 11.06 | 8.78 | 2.96 | 8.25 | 4.63 | 18.79 | 9.51 | 112.20 | 21.67 |
| Camphor | 1032.06 | 171.13 | 64.04 | 15.82 | 10.60 | 5.76 | 2.75 | 3.78 | 3.04 | 20.87 | 14.62 | 124.89 | 41.99 |
| Phoebe | 623.10 | 232.79 | 60.99 | 29.44 | 12.03 | 9.09 | 2.79 | 7.59 | 4.81 | 40.22 | 18.63 | 146.45 | 32.77 |
| Oak | 29202.89 | 169.50 | 47.02 | 17.53 | 9.59 | 6.31 | 2.56 | 4.45 | 2.97 | 23.55 | 14.43 | 117.65 | 27.06 |
| Cyclobalanopsis | 15720.00 | 148.55 | 33.58 | 16.60 | 6.90 | 6.39 | 1.89 | 3.57 | 2.03 | 19.89 | 11.11 | 102.11 | 18.42 |
| Beech | 31.21 | 179.75 | 59.81 | 13.94 | 10.59 | 5.10 | 2.83 | 3.47 | 2.77 | 23.91 | 13.22 | 133.34 | 38.69 |
| Birch | 1597.25 | 154.28 | 49.75 | 15.46 | 9.38 | 5.55 | 2.79 | 3.28 | 2.50 | 20.03 | 15.32 | 109.95 | 27.93 |
| Basswood | 3191.13 | 204.89 | 50.49 | 20.43 | 11.18 | 6.89 | 2.68 | 5.28 | 3.38 | 26.52 | 15.44 | 145.78 | 27.82 |
| Locust | 308.97 | 228.01 | 57.01 | 32.81 | 13.05 | 9.63 | 3.54 | 10.38 | 4.29 | 28.57 | 11.75 | 146.61 | 32.59 |
| Katus | 344.84 | 139.64 | 32.56 | 13.48 | 6.82 | 5.42 | 2.03 | 4.02 | 2.39 | 10.78 | 4.89 | 105.94 | 24.77 |
| Maple | 2257.73 | 149.10 | 35.97 | 15.83 | 6.09 | 6.00 | 1.62 | 2.96 | 1.73 | 15.94 | 8.80 | 108.36 | 24.85 |
| Melia | 33941.82 | 195.28 | 55.53 | 22.63 | 9.14 | 7.58 | 2.22 | 5.11 | 2.80 | 28.53 | 15.05 | 131.42 | 34.42 |
| Chinese toon | 751.23 | 278.07 | 83.25 | 36.63 | 15.82 | 10.11 | 3.36 | 10.67 | 5.63 | 52.76 | 24.55 | 167.89 | 51.97 |
| Elm | 972.00 | 137.97 | 34.51 | 10.69 | 5.98 | 4.72 | 1.84 | 2.62 | 1.81 | 15.35 | 8.56 | 104.59 | 24.67 |
| Ebony | 125.10 | 172.77 | 44.03 | 17.23 | 9.30 | 6.08 | 2.80 | 3.81 | 2.70 | 14.32 | 8.26 | 131.33 | 30.63 |
| Firmiana | 665.06 | 168.80 | 41.57 | 21.72 | 7.83 | 7.42 | 1.92 | 4.74 | 2.28 | 21.00 | 10.08 | 113.91 | 28.75 |
| Other broad-leaved tree species (groups) | 161886.63 | 120.41 | 16.37 | 10.73 | 3.88 | 4.94 | 1.22 | 2.32 | 1.21 | 14.72 | 7.00 | 87.70 | 10.32 |
| Broad-leaved mixed tree species (groups) | 87057.90 | 213.83 | 62.10 | 24.05 | 11.97 | 7.87 | 2.98 | 5.45 | 3.27 | 30.00 | 16.24 | 146.45 | 39.31 |
| Bamboo species | 12421.92 | 118.39 | 9.75 | 17.76 | 1.83 | 4.83 | 0.50 | 4.65 | 2.01 | 6.35 | 2.70 | 84.81 | 10.03 |
| Economic species | 28072.88 | 95.69 | 13.52 | 11.20 | 6.67 | 1.12 | 0.60 | 3.32 | 2.51 | 5.44 | 3.79 | 74.60 | 12.88 |
| Shrub species | 495635.38 | 96.32 | 10.46 | 6.39 | 2.25 | 1.44 | 0.45 | 1.27 | 0.55 | 9.01 | 4.13 | 78.21 | 9.69 |
| Total area | 1432224.21 | 126.01 | 42.86 | 13.09 | 11.11 | 3.38 | 2.68 | 2.65 | 2.38 | 13.30 | 8.68 | 93.59 | 25.24 |

**Table S16** Disturbance matrices in Guizhou. According to Kurz et al. (2009), for all disturbances that involve burning, 90% of the C losses from burned organic matter goes into CO_2_ emissions, the remainder being emitted as CH_4_ (1%) and CO (9%). The amount of N_2_O is estimated as 0.00017 times the amount of CO_2_. RL = regeneration logging, HL = harvest logging, DFA = deforestation for agriculture, FCG = forest conversion to grassland, FCW = forest conversion to waters, DFB = deforestation for built-up land, FDB = forest degradation to bare land, mb = merchantable, f = foliage, o = other wood, cr = coarse roots, fr = fine roots.

| Disturbance type | Pool where C transferred from | Pool where C transferred to | Proportion |
| --- | --- | --- | --- |
| RL | Softwood Biomass_mb | DOM_Medium | 0.1 |
| RL | Softwood Biomass_mb | CO_2_ | 0.045 |
| RL | Softwood Biomass_mb | CH_4_ | 0.0005 |
| RL | Softwood Biomass_mb | CO | 0.0045 |
| RL | Softwood Biomass_mb | Products | 0.85 |
| RL | Softwood Biomass_f | DOM_AG_very_fast | 0.6 |
| RL | Softwood Biomass_f | CO_2_ | 0.36 |
| RL | Softwood Biomass_f | CH_4_ | 0.004 |
| RL | Softwood Biomass_f | CO | 0.036 |
| RL | Softwood Biomass_o | DOM_AG_fast | 0.2 |
| RL | Softwood Biomass_o | CO_2_ | 0.135 |
| RL | Softwood Biomass_o | CH_4_ | 0.0015 |
| RL | Softwood Biomass_o | CO | 0.0135 |
| RL | Softwood Biomass_o | Products | 0.65 |
| RL | Softwood Biomass_cr | DOM_AG_fast | 0.5 |
| RL | Softwood Biomass_cr | DOM_BG_fast | 0.5 |
| RL | Softwood Biomass_fr | DOM_AG_very_fast | 0.15 |
| RL | Softwood Biomass_fr | DOM_BG_very_fast | 0.5 |
| RL | Softwood Biomass_fr | CO_2_ | 0.315 |
| RL | Softwood Biomass_fr | CH_4_ | 0.0035 |
| RL | Softwood Biomass_fr | CO | 0.0315 |
| RL | Hardwood Biomass_mb | DOM_Medium | 0.1 |
| RL | Hardwood Biomass_mb | CO_2_ | 0.045 |
| RL | Hardwood Biomass_mb | CH_4_ | 0.0005 |
| RL | Hardwood Biomass_mb | CO | 0.0045 |
| RL | Hardwood Biomass_mb | Products | 0.85 |
| RL | Hardwood Biomass_f | DOM_AG_very_fast | 0.6 |
| RL | Hardwood Biomass_f | CO_2_ | 0.36 |
| RL | Hardwood Biomass_f | CH_4_ | 0.004 |
| RL | Hardwood Biomass_f | CO | 0.036 |
| RL | Hardwood Biomass_o | DOM_AG_fast | 0.2 |
| RL | Hardwood Biomass_o | CO_2_ | 0.135 |
| RL | Hardwood Biomass_o | CH_4_ | 0.0015 |
| RL | Hardwood Biomass_o | CO | 0.0135 |
| RL | Hardwood Biomass_o | Products | 0.65 |
| RL | Hardwood Biomass_cr | DOM_AG_fast | 0.5 |
| RL | Hardwood Biomass_cr | DOM_BG_fast | 0.5 |
| RL | Hardwood Biomass_fr | DOM_AG_very_fast | 0.15 |
| RL | Hardwood Biomass_fr | DOM_BG_very_fast | 0.5 |
| RL | Hardwood Biomass_fr | CO_2_ | 0.315 |
| RL | Hardwood Biomass_fr | CH_4_ | 0.0035 |
| RL | Hardwood Biomass_fr | CO | 0.0315 |
| RL | DOM_AG_very_fast | DOM_AG_very_fast | 0.3 |
| RL | DOM_AG_very_fast | CO_2_ | 0.63 |
| RL | DOM_AG_very_fast | CH_4_ | 0.007 |
| RL | DOM_AG_very_fast | CO | 0.063 |
| RL | DOM_BG_very_fast | DOM_BG_very_fast | 1 |
| RL | DOM_AG_fast | DOM_AG_fast | 0.3 |
| RL | DOM_AG_fast | CO_2_ | 0.63 |
| RL | DOM_AG_fast | CH_4_ | 0.007 |
| RL | DOM_AG_fast | CO | 0.063 |
| RL | DOM_BG_fast | DOM_BG_fast | 1 |
| RL | DOM_Medium | DOM_Medium | 0.65 |
| RL | DOM_Medium | CO_2_ | 0.315 |
| RL | DOM_Medium | CH_4_ | 0.0035 |
| RL | DOM_Medium | CO | 0.0315 |
| RL | DOM_AG_slow | DOM_AG_slow | 1 |
| RL | DOM_BG_slow | DOM_BG_slow | 1 |
| RL | Softwood DOM_Snag_stem | DOM_Medium | 0.65 |
| RL | Softwood DOM_Snag_stem | CO_2_ | 0.315 |
| RL | Softwood DOM_Snag_stem | CH_4_ | 0.0035 |
| RL | Softwood DOM_Snag_stem | CO | 0.0315 |
| RL | Softwood DOM_Snag_branch | DOM_AG_fast | 0.3 |
| RL | Softwood DOM_Snag_branch | CO_2_ | 0.63 |
| RL | Softwood DOM_Snag_branch | CH_4_ | 0.007 |
| RL | Softwood DOM_Snag_branch | CO | 0.063 |
| RL | Hardwood DOM_Snag_stem | DOM_Medium | 0.65 |
| RL | Hardwood DOM_Snag_stem | CO_2_ | 0.315 |
| RL | Hardwood DOM_Snag_stem | CH_4_ | 0.0035 |
| RL | Hardwood DOM_Snag_stem | CO | 0.0315 |
| RL | Hardwood DOM_Snag_branch | DOM_AG_fast | 0.3 |
| RL | Hardwood DOM_Snag_branch | CO_2_ | 0.63 |
| RL | Hardwood DOM_Snag_branch | CH_4_ | 0.007 |
| RL | Hardwood DOM_Snag_branch | CO | 0.063 |
| HL | Softwood Biomass_mb | DOM_Medium | 0.1 |
| HL | Softwood Biomass_mb | CO_2_ | 0.045 |
| HL | Softwood Biomass_mb | CH_4_ | 0.0005 |
| HL | Softwood Biomass_mb | CO | 0.0045 |
| HL | Softwood Biomass_mb | Products | 0.85 |
| HL | Softwood Biomass_f | DOM_AG_very_fast | 0.6 |
| HL | Softwood Biomass_f | CO_2_ | 0.36 |
| HL | Softwood Biomass_f | CH_4_ | 0.004 |
| HL | Softwood Biomass_f | CO | 0.036 |
| HL | Softwood Biomass_o | DOM_AG_fast | 0.4 |
| HL | Softwood Biomass_o | CO_2_ | 0.27 |
| HL | Softwood Biomass_o | CH_4_ | 0.003 |
| HL | Softwood Biomass_o | CO | 0.027 |
| HL | Softwood Biomass_o | Products | 0.3 |
| HL | Softwood Biomass_cr | DOM_AG_fast | 0.5 |
| HL | Softwood Biomass_cr | DOM_BG_fast | 0.5 |
| HL | Softwood Biomass_fr | DOM_AG_very_fast | 0.15 |
| HL | Softwood Biomass_fr | DOM_BG_very_fast | 0.5 |
| HL | Softwood Biomass_fr | CO_2_ | 0.315 |
| HL | Softwood Biomass_fr | CH_4_ | 0.0035 |
| HL | Softwood Biomass_fr | CO | 0.0315 |
| HL | Hardwood Biomass_mb | DOM_Medium | 0.1 |
| HL | Hardwood Biomass_mb | CO_2_ | 0.045 |
| HL | Hardwood Biomass_mb | CH_4_ | 0.0005 |
| HL | Hardwood Biomass_mb | CO | 0.0045 |
| HL | Hardwood Biomass_mb | Products | 0.85 |
| HL | Hardwood Biomass_f | DOM_AG_very_fast | 0.6 |
| HL | Hardwood Biomass_f | CO_2_ | 0.36 |
| HL | Hardwood Biomass_f | CH_4_ | 0.004 |
| HL | Hardwood Biomass_f | CO | 0.036 |
| HL | Hardwood Biomass_o | DOM_AG_fast | 0.4 |
| HL | Hardwood Biomass_o | CO_2_ | 0.27 |
| HL | Hardwood Biomass_o | CH_4_ | 0.003 |
| HL | Hardwood Biomass_o | CO | 0.027 |
| HL | Hardwood Biomass_o | Products | 0.3 |
| HL | Hardwood Biomass_cr | DOM_AG_fast | 0.5 |
| HL | Hardwood Biomass_cr | DOM_BG_fast | 0.5 |
| HL | Hardwood Biomass_fr | DOM_AG_very_fast | 0.15 |
| HL | Hardwood Biomass_fr | DOM_BG_very_fast | 0.5 |
| HL | Hardwood Biomass_fr | CO_2_ | 0.315 |
| HL | Hardwood Biomass_fr | CH_4_ | 0.0035 |
| HL | Hardwood Biomass_fr | CO | 0.0315 |
| HL | DOM_AG_very_fast | DOM_AG_very_fast | 0.3 |
| HL | DOM_AG_very_fast | CO_2_ | 0.63 |
| HL | DOM_AG_very_fast | CH_4_ | 0.007 |
| HL | DOM_AG_very_fast | CO | 0.063 |
| HL | DOM_BG_very_fast | DOM_BG_very_fast | 1 |
| HL | DOM_AG_fast | DOM_AG_fast | 0.3 |
| HL | DOM_AG_fast | CO_2_ | 0.63 |
| HL | DOM_AG_fast | CH_4_ | 0.007 |
| HL | DOM_AG_fast | CO | 0.063 |
| HL | DOM_BG_fast | DOM_BG_fast | 1 |
| HL | DOM_Medium | DOM_Medium | 0.65 |
| HL | DOM_Medium | CO_2_ | 0.315 |
| HL | DOM_Medium | CH_4_ | 0.0035 |
| HL | DOM_Medium | CO | 0.0315 |
| HL | DOM_AG_slow | DOM_AG_slow | 1 |
| HL | DOM_BG_slow | DOM_BG_slow | 1 |
| HL | Softwood DOM_Snag_stem | DOM_Medium | 0.65 |
| HL | Softwood DOM_Snag_stem | CO_2_ | 0.315 |
| HL | Softwood DOM_Snag_stem | CH_4_ | 0.0035 |
| HL | Softwood DOM_Snag_stem | CO | 0.0315 |
| HL | Softwood DOM_Snag_branch | DOM_AG_fast | 0.3 |
| HL | Softwood DOM_Snag_branch | CO_2_ | 0.63 |
| HL | Softwood DOM_Snag_branch | CH_4_ | 0.007 |
| HL | Softwood DOM_Snag_branch | CO | 0.063 |
| HL | Hardwood DOM_Snag_stem | DOM_Medium | 0.65 |
| HL | Hardwood DOM_Snag_stem | CO_2_ | 0.315 |
| HL | Hardwood DOM_Snag_stem | CH_4_ | 0.0035 |
| HL | Hardwood DOM_Snag_stem | CO | 0.0315 |
| HL | Hardwood DOM_Snag_branch | DOM_AG_fast | 0.3 |
| HL | Hardwood DOM_Snag_branch | CO_2_ | 0.63 |
| HL | Hardwood DOM_Snag_branch | CH_4_ | 0.007 |
| HL | Hardwood DOM_Snag_branch | CO | 0.063 |
| DFA | Softwood Biomass_mb | DOM_Medium | 0.2 |
| DFA | Softwood Biomass_mb | CO_2_ | 0.09 |
| DFA | Softwood Biomass_mb | CH_4_ | 0.001 |
| DFA | Softwood Biomass_mb | CO | 0.009 |
| DFA | Softwood Biomass_mb | Products | 0.7 |
| DFA | Softwood Biomass_f | DOM_AG_very_fast | 0.6 |
| DFA | Softwood Biomass_f | CO_2_ | 0.36 |
| DFA | Softwood Biomass_f | CH_4_ | 0.004 |
| DFA | Softwood Biomass_f | CO | 0.036 |
| DFA | Softwood Biomass_o | DOM_AG_fast | 0.2 |
| DFA | Softwood Biomass_o | CO_2_ | 0.27 |
| DFA | Softwood Biomass_o | CH_4_ | 0.003 |
| DFA | Softwood Biomass_o | CO | 0.027 |
| DFA | Softwood Biomass_o | Products | 0.5 |
| DFA | Softwood Biomass_cr | DOM_AG_fast | 0.3 |
| DFA | Softwood Biomass_cr | CO_2_ | 0.63 |
| DFA | Softwood Biomass_cr | CH_4_ | 0.007 |
| DFA | Softwood Biomass_cr | CO | 0.063 |
| DFA | Softwood Biomass_fr | DOM_BG_very_fast | 0.5 |
| DFA | Softwood Biomass_fr | CO_2_ | 0.45 |
| DFA | Softwood Biomass_fr | CH_4_ | 0.005 |
| DFA | Softwood Biomass_fr | CO | 0.045 |
| DFA | Hardwood Biomass_mb | DOM_Medium | 0.2 |
| DFA | Hardwood Biomass_mb | CO_2_ | 0.09 |
| DFA | Hardwood Biomass_mb | CH_4_ | 0.001 |
| DFA | Hardwood Biomass_mb | CO | 0.009 |
| DFA | Hardwood Biomass_mb | Products | 0.7 |
| DFA | Hardwood Biomass_f | DOM_AG_very_fast | 0.6 |
| DFA | Hardwood Biomass_f | CO_2_ | 0.36 |
| DFA | Hardwood Biomass_f | CH_4_ | 0.004 |
| DFA | Hardwood Biomass_f | CO | 0.036 |
| DFA | Hardwood Biomass_o | DOM_AG_fast | 0.2 |
| DFA | Hardwood Biomass_o | CO_2_ | 0.27 |
| DFA | Hardwood Biomass_o | CH_4_ | 0.003 |
| DFA | Hardwood Biomass_o | CO | 0.027 |
| DFA | Hardwood Biomass_o | Products | 0.5 |
| DFA | Hardwood Biomass_cr | DOM_AG_fast | 0.3 |
| DFA | Hardwood Biomass_cr | CO_2_ | 0.63 |
| DFA | Hardwood Biomass_cr | CH_4_ | 0.007 |
| DFA | Hardwood Biomass_cr | CO | 0.063 |
| DFA | Hardwood Biomass_fr | DOM_BG_very_fast | 0.5 |
| DFA | Hardwood Biomass_fr | CO_2_ | 0.45 |
| DFA | Hardwood Biomass_fr | CH_4_ | 0.005 |
| DFA | Hardwood Biomass_fr | CO | 0.045 |
| DFA | DOM_AG_very_fast | DOM_BG_very_fast | 0.3 |
| DFA | DOM_AG_very_fast | CO_2_ | 0.63 |
| DFA | DOM_AG_very_fast | CH_4_ | 0.007 |
| DFA | DOM_AG_very_fast | CO | 0.063 |
| DFA | DOM_BG_very_fast | DOM_BG_very_fast | 1 |
| DFA | DOM_AG_fast | DOM_AG_fast | 0.3 |
| DFA | DOM_AG_fast | CO_2_ | 0.63 |
| DFA | DOM_AG_fast | CH_4_ | 0.007 |
| DFA | DOM_AG_fast | CO | 0.063 |
| DFA | DOM_BG_fast | DOM_AG_fast | 0.3 |
| DFA | DOM_BG_fast | CO_2_ | 0.63 |
| DFA | DOM_BG_fast | CH_4_ | 0.007 |
| DFA | DOM_BG_fast | CO | 0.063 |
| DFA | DOM_Medium | DOM_Medium | 0.3 |
| DFA | DOM_Medium | CO_2_ | 0.63 |
| DFA | DOM_Medium | CH_4_ | 0.007 |
| DFA | DOM_Medium | CO | 0.063 |
| DFA | DOM_AG_slow | DOM_BG_slow | 1 |
| DFA | DOM_BG_slow | DOM_BG_slow | 1 |
| DFA | Softwood DOM_Snag_stem | DOM_Medium | 0.3 |
| DFA | Softwood DOM_Snag_stem | CO_2_ | 0.63 |
| DFA | Softwood DOM_Snag_stem | CH_4_ | 0.007 |
| DFA | Softwood DOM_Snag_stem | CO | 0.063 |
| DFA | Softwood DOM_Snag_branch | DOM_AG_fast | 0.3 |
| DFA | Softwood DOM_Snag_branch | CO_2_ | 0.63 |
| DFA | Softwood DOM_Snag_branch | CH_4_ | 0.007 |
| DFA | Softwood DOM_Snag_branch | CO | 0.063 |
| DFA | Hardwood DOM_Snag_stem | DOM_Medium | 0.3 |
| DFA | Hardwood DOM_Snag_stem | CO_2_ | 0.63 |
| DFA | Hardwood DOM_Snag_stem | CH_4_ | 0.007 |
| DFA | Hardwood DOM_Snag_stem | CO | 0.063 |
| DFA | Hardwood DOM_Snag_branch | DOM_AG_fast | 0.3 |
| DFA | Hardwood DOM_Snag_branch | CO_2_ | 0.63 |
| DFA | Hardwood DOM_Snag_branch | CH_4_ | 0.007 |
| DFA | Hardwood DOM_Snag_branch | CO | 0.063 |
| FCG | Softwood Biomass_mb | Softwood DOM_Snag_stem | 0.3 |
| FCG | Softwood Biomass_mb | Products | 0.7 |
| FCG | Softwood Biomass_f | DOM_BG_very_fast | 1 |
| FCG | Softwood Biomass_o | DOM_AG_fast | 0.2 |
| FCG | Softwood Biomass_o | Softwood DOM_Snag_branch | 0.3 |
| FCG | Softwood Biomass_o | Products | 0.5 |
| FCG | Softwood Biomass_cr | DOM_AG_fast | 1 |
| FCG | Softwood Biomass_fr | DOM_BG_very_fast | 1 |
| FCG | Hardwood Biomass_mb | Hardwood DOM_Snag_stem | 0.3 |
| FCG | Hardwood Biomass_mb | Products | 0.7 |
| FCG | Hardwood Biomass_f | DOM_BG_very_fast | 1 |
| FCG | Hardwood Biomass_o | DOM_AG_fast | 0.2 |
| FCG | Hardwood Biomass_o | Hardwood DOM_Snag_branch | 0.3 |
| FCG | Hardwood Biomass_o | Products | 0.5 |
| FCG | Hardwood Biomass_cr | DOM_AG_fast | 1 |
| FCG | Hardwood Biomass_fr | DOM_BG_very_fast | 1 |
| FCG | DOM_AG_very_fast | DOM_BG_very_fast | 1 |
| FCG | DOM_BG_very_fast | DOM_BG_very_fast | 1 |
| FCG | DOM_AG_fast | DOM_AG_fast | 1 |
| FCG | DOM_BG_fast | DOM_AG_fast | 1 |
| FCG | DOM_Medium | DOM_Medium | 1 |
| FCG | DOM_AG_slow | DOM_BG_slow | 1 |
| FCG | DOM_BG_slow | DOM_BG_slow | 1 |
| FCG | Softwood DOM_Snag_stem | DOM_Medium | 1 |
| FCG | Softwood DOM_Snag_branch | DOM_AG_fast | 1 |
| FCG | Hardwood DOM_Snag_stem | DOM_Medium | 1 |
| FCG | Hardwood DOM_Snag_branch | DOM_AG_fast | 1 |
| FCW | Softwood Biomass_mb | DOM_Medium | 0.15 |
| FCW | Softwood Biomass_mb | Products | 0.85 |
| FCW | Softwood Biomass_f | DOM_AG_very_fast | 1 |
| FCW | Softwood Biomass_o | DOM_AG_fast | 1 |
| FCW | Softwood Biomass_cr | DOM_AG_fast | 0.5 |
| FCW | Softwood Biomass_cr | DOM_BG_fast | 0.5 |
| FCW | Softwood Biomass_fr | DOM_BG_very_fast | 1 |
| FCW | Hardwood Biomass_mb | DOM_Medium | 0.15 |
| FCW | Hardwood Biomass_mb | Products | 0.85 |
| FCW | Hardwood Biomass_f | DOM_AG_very_fast | 1 |
| FCW | Hardwood Biomass_o | DOM_AG_fast | 1 |
| FCW | Hardwood Biomass_cr | DOM_AG_fast | 0.5 |
| FCW | Hardwood Biomass_cr | DOM_BG_fast | 0.5 |
| FCW | Hardwood Biomass_fr | DOM_BG_very_fast | 1 |
| FCW | DOM_AG_very_fast | DOM_BG_very_fast | 1 |
| FCW | DOM_BG_very_fast | DOM_BG_very_fast | 1 |
| FCW | DOM_AG_fast | DOM_BG_fast | 1 |
| FCW | DOM_BG_fast | DOM_BG_fast | 1 |
| FCW | DOM_Medium | DOM_Medium | 1 |
| FCW | DOM_AG_slow | DOM_AG_slow | 1 |
| FCW | DOM_BG_slow | DOM_BG_slow | 1 |
| FCW | Softwood DOM_Snag_stem | DOM_Medium | 0.5 |
| FCW | Softwood DOM_Snag_stem | Products | 0.5 |
| FCW | Softwood DOM_Snag_branch | DOM_AG_fast | 1 |
| FCW | Hardwood DOM_Snag_stem | DOM_Medium | 0.5 |
| FCW | Hardwood DOM_Snag_stem | Products | 0.5 |
| FCW | Hardwood DOM_Snag_branch | DOM_AG_fast | 1 |
| DFB | Softwood Biomass_mb | DOM_Medium | 0.1 |
| DFB | Softwood Biomass_mb | CO_2_ | 0.045 |
| DFB | Softwood Biomass_mb | CH_4_ | 0.0005 |
| DFB | Softwood Biomass_mb | CO | 0.0045 |
| DFB | Softwood Biomass_mb | Products | 0.85 |
| DFB | Softwood Biomass_f | DOM_AG_very_fast | 0.2 |
| DFB | Softwood Biomass_f | CO_2_ | 0.09 |
| DFB | Softwood Biomass_f | CH_4_ | 0.001 |
| DFB | Softwood Biomass_f | CO | 0.009 |
| DFB | Softwood Biomass_f | Products | 0.7 |
| DFB | Softwood Biomass_o | DOM_AG_fast | 0.1 |
| DFB | Softwood Biomass_o | CO_2_ | 0.18 |
| DFB | Softwood Biomass_o | CH_4_ | 0.002 |
| DFB | Softwood Biomass_o | CO | 0.018 |
| DFB | Softwood Biomass_o | Products | 0.7 |
| DFB | Softwood Biomass_cr | DOM_AG_fast | 0.15 |
| DFB | Softwood Biomass_cr | CO_2_ | 0.135 |
| DFB | Softwood Biomass_cr | CH_4_ | 0.0015 |
| DFB | Softwood Biomass_cr | CO | 0.0135 |
| DFB | Softwood Biomass_cr | Products | 0.7 |
| DFB | Softwood Biomass_fr | DOM_BG_very_fast | 0.2 |
| DFB | Softwood Biomass_fr | CO_2_ | 0.09 |
| DFB | Softwood Biomass_fr | CH_4_ | 0.001 |
| DFB | Softwood Biomass_fr | CO | 0.009 |
| DFB | Softwood Biomass_fr | Products | 0.7 |
| DFB | Hardwood Biomass_mb | DOM_Medium | 0.1 |
| DFB | Hardwood Biomass_mb | CO_2_ | 0.045 |
| DFB | Hardwood Biomass_mb | CH_4_ | 0.0005 |
| DFB | Hardwood Biomass_mb | CO | 0.0045 |
| DFB | Hardwood Biomass_mb | Products | 0.85 |
| DFB | Hardwood Biomass_f | DOM_AG_very_fast | 0.2 |
| DFB | Hardwood Biomass_f | CO_2_ | 0.09 |
| DFB | Hardwood Biomass_f | CH_4_ | 0.001 |
| DFB | Hardwood Biomass_f | CO | 0.009 |
| DFB | Hardwood Biomass_f | Products | 0.7 |
| DFB | Hardwood Biomass_o | DOM_AG_fast | 0.1 |
| DFB | Hardwood Biomass_o | CO_2_ | 0.18 |
| DFB | Hardwood Biomass_o | CH_4_ | 0.002 |
| DFB | Hardwood Biomass_o | CO | 0.018 |
| DFB | Hardwood Biomass_o | Products | 0.7 |
| DFB | Hardwood Biomass_cr | DOM_AG_fast | 0.15 |
| DFB | Hardwood Biomass_cr | CO_2_ | 0.135 |
| DFB | Hardwood Biomass_cr | CH_4_ | 0.0015 |
| DFB | Hardwood Biomass_cr | CO | 0.0135 |
| DFB | Hardwood Biomass_cr | Products | 0.7 |
| DFB | Hardwood Biomass_fr | DOM_BG_very_fast | 0.2 |
| DFB | Hardwood Biomass_fr | CO_2_ | 0.09 |
| DFB | Hardwood Biomass_fr | CH_4_ | 0.001 |
| DFB | Hardwood Biomass_fr | CO | 0.009 |
| DFB | Hardwood Biomass_fr | Products | 0.7 |
| DFB | DOM_AG_very_fast | DOM_BG_very_fast | 0.3 |
| DFB | DOM_AG_very_fast | CO_2_ | 0.18 |
| DFB | DOM_AG_very_fast | CH_4_ | 0.002 |
| DFB | DOM_AG_very_fast | CO | 0.018 |
| DFB | DOM_AG_very_fast | Products | 0.5 |
| DFB | DOM_BG_very_fast | DOM_BG_very_fast | 1 |
| DFB | DOM_AG_fast | DOM_AG_fast | 0.15 |
| DFB | DOM_AG_fast | CO_2_ | 0.225 |
| DFB | DOM_AG_fast | CH_4_ | 0.0025 |
| DFB | DOM_AG_fast | CO | 0.0225 |
| DFB | DOM_AG_fast | Products | 0.6 |
| DFB | DOM_BG_fast | DOM_AG_fast | 0.15 |
| DFB | DOM_BG_fast | CO_2_ | 0.225 |
| DFB | DOM_BG_fast | CH_4_ | 0.0025 |
| DFB | DOM_BG_fast | CO | 0.0225 |
| DFB | DOM_BG_fast | Products | 0.6 |
| DFB | DOM_Medium | DOM_Medium | 0.1 |
| DFB | DOM_Medium | CO_2_ | 0.225 |
| DFB | DOM_Medium | CH_4_ | 0.0025 |
| DFB | DOM_Medium | CO | 0.0225 |
| DFB | DOM_Medium | Products | 0.65 |
| DFB | DOM_AG_slow | DOM_BG_slow | 1 |
| DFB | DOM_BG_slow | DOM_BG_slow | 1 |
| DFB | Softwood DOM_Snag_stem | DOM_Medium | 0.1 |
| DFB | Softwood DOM_Snag_stem | CO_2_ | 0.045 |
| DFB | Softwood DOM_Snag_stem | CH_4_ | 0.0005 |
| DFB | Softwood DOM_Snag_stem | CO | 0.0045 |
| DFB | Softwood DOM_Snag_stem | Products | 0.85 |
| DFB | Softwood DOM_Snag_branch | DOM_AG_fast | 0.1 |
| DFB | Softwood DOM_Snag_branch | CO_2_ | 0.18 |
| DFB | Softwood DOM_Snag_branch | CH_4_ | 0.002 |
| DFB | Softwood DOM_Snag_branch | CO | 0.018 |
| DFB | Softwood DOM_Snag_branch | Products | 0.7 |
| DFB | Hardwood DOM_Snag_stem | DOM_Medium | 0.1 |
| DFB | Hardwood DOM_Snag_stem | CO_2_ | 0.045 |
| DFB | Hardwood DOM_Snag_stem | CH_4_ | 0.0005 |
| DFB | Hardwood DOM_Snag_stem | CO | 0.0045 |
| DFB | Hardwood DOM_Snag_stem | Products | 0.85 |
| DFB | Hardwood DOM_Snag_branch | DOM_AG_fast | 0.1 |
| DFB | Hardwood DOM_Snag_branch | CO_2_ | 0.18 |
| DFB | Hardwood DOM_Snag_branch | CH_4_ | 0.002 |
| DFB | Hardwood DOM_Snag_branch | CO | 0.018 |
| DFB | Hardwood DOM_Snag_branch | Products | 0.7 |
| FDB | Softwood Biomass_mb | DOM_Medium | 0.2 |
| FDB | Softwood Biomass_mb | Products | 0.8 |
| FDB | Softwood Biomass_f | DOM_AG_very_fast | 1 |
| FDB | Softwood Biomass_o | DOM_AG_fast | 0.4 |
| FDB | Softwood Biomass_o | Products | 0.6 |
| FDB | Softwood Biomass_cr | DOM_AG_fast | 0.5 |
| FDB | Softwood Biomass_cr | DOM_BG_fast | 0.5 |
| FDB | Softwood Biomass_fr | DOM_AG_very_fast | 0.5 |
| FDB | Softwood Biomass_fr | DOM_BG_very_fast | 0.5 |
| FDB | Hardwood Biomass_mb | DOM_Medium | 0.2 |
| FDB | Hardwood Biomass_mb | Products | 0.8 |
| FDB | Hardwood Biomass_f | DOM_AG_very_fast | 1 |
| FDB | Hardwood Biomass_o | DOM_AG_fast | 0.4 |
| FDB | Hardwood Biomass_o | Products | 0.6 |
| FDB | Hardwood Biomass_cr | DOM_AG_fast | 0.5 |
| FDB | Hardwood Biomass_cr | DOM_BG_fast | 0.5 |
| FDB | Hardwood Biomass_fr | DOM_AG_very_fast | 0.5 |
| FDB | Hardwood Biomass_fr | DOM_BG_very_fast | 0.5 |
| FDB | DOM_AG_very_fast | DOM_AG_very_fast | 1 |
| FDB | DOM_BG_very_fast | DOM_BG_very_fast | 1 |
| FDB | DOM_AG_fast | DOM_AG_fast | 1 |
| FDB | DOM_BG_fast | DOM_BG_fast | 1 |
| FDB | DOM_Medium | DOM_Medium | 1 |
| FDB | DOM_AG_slow | DOM_AG_slow | 1 |
| FDB | DOM_BG_slow | DOM_BG_slow | 1 |
| FDB | Softwood DOM_Snag_stem | DOM_Medium | 1 |
| FDB | Softwood DOM_Snag_branch | DOM_AG_fast | 1 |
| FDB | Hardwood DOM_Snag_stem | DOM_Medium | 1 |
| FDB | Hardwood DOM_Snag_branch | DOM_AG_fast | 1 |


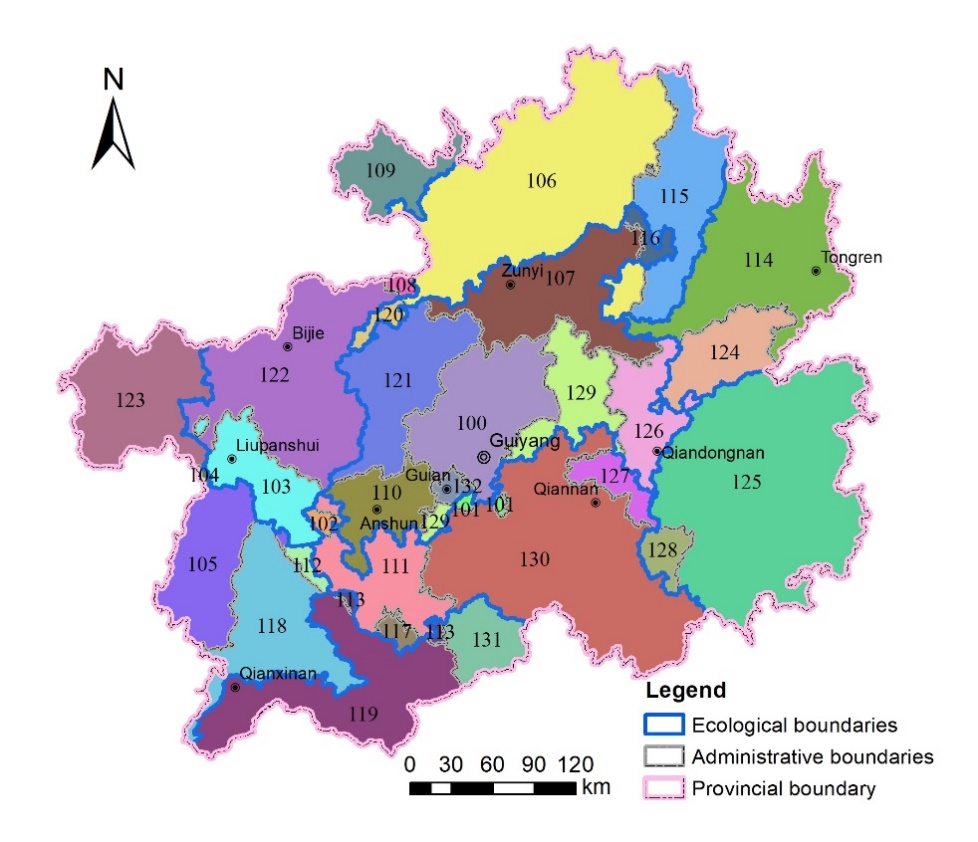


**Fig. S1** Guizhou spatial unit (SPU) distribution

**Part V**

Annual temperature trend during 1990–2016 in Guizhou

**Fig. S2** Interannual variation of annual temperature in Guizhou from 1990 to 2016

**References**

Bai, B., Jiao, S., Chen, D., 2017. The Biomass and Carbon Fixation of 38 Tree Species in Northern and Central Region of Henan Province. Journal of West China Forestry Science. 46(1), 79-84. https://doi.org/10.16473/j.cnki.xblykx1972.2017.01.015 (in Chinese).

Feng, Y., 2014. The Research on Carbon Budget of Forest Ecosystem in Pu'er Region of Yunnan Province Based on CBM Model. Chinese Academy of Forestry, Beijing (in Chinese).

Fu, T., 2013. Carbon Estimation of Main Forest Ecosystem in Three Gorges Reservoir Area using CBM-CFS3. Chinese Academy of Forestry, Beijing (in Chinese).

Guizhou Forestry Bureau. 2015. Detailed Rules for the Implementation of Fourth Forest Resources Planning and Design Survey of Guizhou Province. Guizhou Forestry Bureau, Guiyang, Guizhou (in Chinese).

Guizhou Forestry Bureau. 2009. Technical Proposal for Annual Forest Harvesting Limit During the 12th Five-Year Plan Period in Guizhou Province. Guizhou Forestry Bureau, Guiyang, Guizhou (in Chinese).

Guizhou Provincial Development and Reform Commission (GPDRC). 2017. Guidelines for Compiling Guizhou Greenhouse Gas Inventory at City- (Prefecture-) Level (Trial). GPDRC, Guiyang, Guizhou (in Chinese).

He, H., He, R., Duan, X., Chen, X., 2007a. Study on Carbon Storage in Main Afforestation Tree Species of the Second Forest Zone around Guiyang City. Journal of Anhui Agricultural Sciences. 35(32), 10270-10271. https://doi.org/10.13989/j.cnki.0517-6611.2007.32.066 (in Chinese).

He, Y., Fei, S., Jiang, J., Chen, X., Yu, Y., Tang, S., Zhu, W., 2007b. The Spatial Distribution of Organic Carbon in Phyllostachys pubescens and Pleioblastus amarus in Changning County. Journal of Sichuan Forestry Science and Technology. 28(5), 10-14. https://doi.org/10.3969/j.issn.1003-5508.2007.05.003 (in Chinese).

Hou, F., Wang, K., Song, Y., Li, J., Ai, Z., Chen, X., 2018. Carbon storage and distribution in typical forest ecosystems in subalpine of middle Yunnan Province. Ecology and Environmental Sciences. 27(10), 1825-1835. https://doi.org/10.16258/j.cnki.1674-5906.2018.10.006 (in Chinese).

Huang, J., Huang, L., Lin, Z., Chen, G., 2010. Controlling Factors of Litter Decomposition Rate in China's Forests. Journal of Subtropical Resources and Environment. 5(3), 56-63. https://doi.org/10.3969/j.issn.1673-7105.2010.03.008 (in Chinese).

Huang, X., Zhou, Y., Zhang, Z., 2017. Distribution Characteristics of Soil Organic Carbon Under Different Land Uses in a Karst Rocky Desertification Area. J Soil Water Conserv. 31(5), 215-221. https://doi.org/10.13870/j.cnki.stbcxb.2017.05.034 (in Chinese).

Kurz, W.A., Dymond, C.C., White, T.M., Stinson, G., Shaw, C.H., Rampley, G.J., Smyth, C., Simpson, B.N., Neilson, E.T., Trofymow, J.A., Metsaranta, J., Apps, M.J., 2009. CBM-CFS3: A model of carbon-dynamics in forestry and land-use change implementing IPCC standards. Ecol Model. 220(4), 480-504. https://doi.org/10.1016/j.ecolmodel.2008.10.018.

Li, H., 2017. Carbon Storage and its Distribution in Camellia oleifera Plantations at  Different  Stand Ages in North Guangxi. Hunan Agricultural Sciences. (5), 53-55. https://doi.org/10.16498/j.cnki.hnnykx.2017.005.015 (in Chinese).

Luo, Y., Wang, X., Zhang, X., Lu, F., 2013. Biomass and Its Allocation of Forest Ecosystems in China. China Forestry Publishing House, Beijing (in Chinese).

National Forestry Administration. 2005. Code for Forest Harvesting (LY/T 1646-2005). National Forestry Administration, Beijing (in Chinese).

Pu, H., Chen, D., Yang, M., Gao, L., Liu, C., Wang, X., 1988. An analysis on the characteristics of the regional ecosystem of Guizhou province. Acta Ecologica Sinica. 8(4), 298-303 (in Chinese).

Shen, X., 2017. Research on Biomass and carbon storage of Castanoposis eyrei natural forest in Subtropiss. Central South University of Forestry and Technology, Changsha, Hunan (in Chinese).

Smyth, C.E., Trofymow, J.A., Kurz, W.A., 2010. Decreasing uncertainty in CBM-CFS3 estimates of forest soil carbon sources and sinks through use of long-term data from the Canadian Intersite Decomposition Experiment. Pacific Forestry Centre, Canadian Forest Service, Victoria.

Tang, X., 2007. Carbon storage of forest vegetation and spatial distribution in Sichuan Province. Sichuan Agricultural University, Chengdu, Sichuan (in Chinese).

Tang Y, Shao Q, Shi T, Wu G., 2021. Developing Growth Models of Stand Volume for Subtropical Forests in Karst Areas: A Case Study in the Guizhou Plateau. Forests. 12: 83. http://doi.org/10.3390/f12010083.

The People's Republic of China (PRC), 2013. The People's Republic of China Second National Communication on Climate Change. Department of Climate Change, National Development and Reform Commission of China, Beijing (in Chinese).

Wang, J., Wang, X., Yue, C., Cheng, F., Xu, T., Cheng, P., Wang, X., Gao, Y., 2012. Carbon content rate in dominant species of four forest types in Shangri-la, northwest Yunnan province. Ecology and Environmental Sciences. 21(4), 613-619. https://doi.org/10.16258/j.cnki.1674-5906.2012.04.010 (in Chinese).

Wang, L., Li, S., Zheng, Y., Wang, B., 2016. Initial Estimation of Carbon Sinks from Sustainable Management of Rare Forests in Hubei Province. Forest Resources Management. (3), 135-139. https://doi.org/10.13466/j.cnki.lyzygl.2016.03.024 (in Chinese).

Wen, W., Tan, Y., Shi, Z., Peng, Y., 2015. Biomass and Carbon Storage and Their Spatial Distribution of Economic Forest in Shenzhen City. Journal of West China Forestry Science. 44(3), 90-96. https://doi.org/10.16473/j.cnki.xblykx1972.2015.03.014 (in Chinese).

Wu, Q., 1983. Types and Stand Structures of Phyllostachys Pubescens in Guizhou Province. Journal of Bamboo Research. 2(1), 112-124 (in Chinese).

Xu, G., 2010. A Brief Study on Guizhou's Historical Change of Forest and Vegetation and its Consequences. Journal of Guizhou University for Nationalities (Philosophy and Social Science). (5), 69-73. https://doi.org/10.3969/j.issn.1003-6644.2010.05.019 (in Chinese).

Yang, X., 2015. Carbon Content of Common Forest Types in Southeastern Guizhou Province. Guizhou Forestry Science and Technology. 43(3), 9-14. https://doi.org/10.16709/j.cnki.gzlykj.2015.03.007 (in Chinese).

Zhou, T., Shi, P., Jia, G., Li, X., Yiqi, L., 2010. Spatial patterns of ecosystem carbon residence time in Chinese forests. Science China Earth Sciences. 40(5), 632-644. https://doi.org/10.1007/s11430-010-0061-8 (in Chinese).

1. The values of parameters which don’t list here are the same as the CBM-CFS3 default parameter values. [↑](#footnote-ref-1)
